# Biological Activity *In Vitro*, Absorption, BBB Penetration, and Tolerability of Nanoformulation of BT44:RET Agonist with Disease-Modifying Potential for the Treatment of Neurodegeneration

**DOI:** 10.1021/acs.biomac.2c00761

**Published:** 2022-10-11

**Authors:** Malik Salman Haider, Arun Kumar Mahato, Anastasiia Kotliarova, Stefan Forster, Bettina Böttcher, Philipp Stahlhut, Yulia Sidorova, Robert Luxenhofer

**Affiliations:** † Functional Polymer Materials, Chair for Advanced Materials Synthesis, Institute for Functional Materials and Biofabrication, Department of Chemistry and Pharmacy, Julius-Maximilians-University Würzburg, Röntgenring 11, 97070Würzburg, Germany; ‡ Laboratory of Molecular Neuroscience, Institute of Biotechnology, HiLIFE, University of Helsinki, 00014Helsinki, Finland; § Biocenter and Rudolf Virchow Centre, Julius-Maximilians-University Würzburg, Haus D15, Josef-Schneider-Strasse 2, 97080Würzburg, Germany; ∥ Department of Functional Materials in Medicine and Dentistry, Institute of Functional Materials and Biofabrication and Bavarian Polymer Institute, Julius-Maximilians-University Würzburg, Pleicherwall 2, 97070Würzburg, Germany; ⊥ Soft Matter Chemistry, Department of Chemistry, and Helsinki Institute of Sustainability Science, Faculty of Science, University of Helsinki, PB 55-00014Helsinki, Finland; △ University Hospital of Würzburg, Department of Ophthalmology, Josef-Schneider-Street 11, D-97080Würzburg, Germany

## Abstract

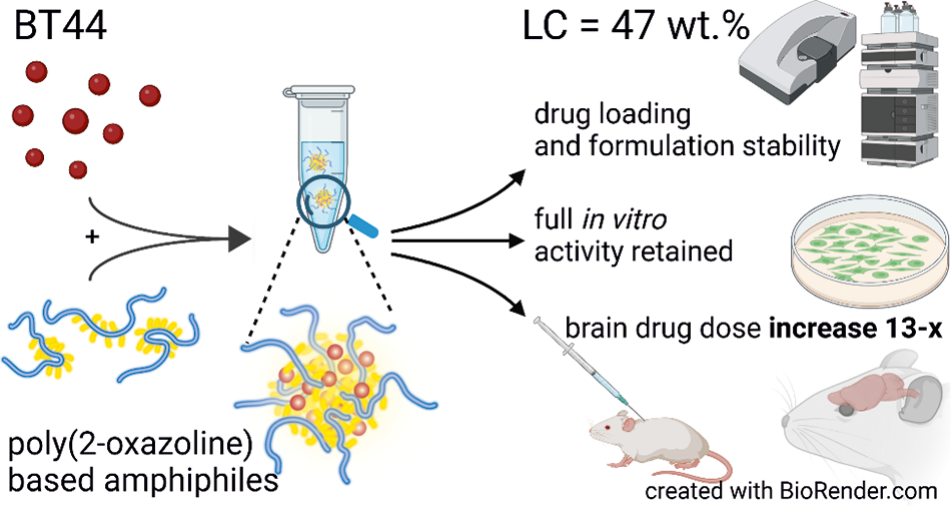

BT44 is a novel, second-generation glial cell line-derived neurotropic factor mimetic with improved biological activity and is a lead compound for the treatment of neurodegenerative disorders. Like many other small molecules, it suffers from intrinsic poor aqueous solubility, posing significant hurdles at various levels for its preclinical development and clinical translation. Herein, we report a poly(2-oxazoline)s (POx)-based BT44 micellar nanoformulation with an ultrahigh drug-loading capacity of 47 wt %. The BT44 nanoformulation was comprehensively characterized by ^1^H NMR spectroscopy, differential scanning calorimetry (DSC), powder X-ray diffraction (XRD), dynamic light scattering (DLS), and cryo-transmission/scanning electron microscopy (cryo-TEM/SEM). The DSC, XRD, and redispersion studies collectively confirmed that the BT44 formulation can be stored as a lyophilized powder and can be redispersed upon need. The DLS suggested that the redispersed formulation is suitable for parenteral administration (*D*_h_ ≈ 70 nm). The cryo-TEM measurements showed the presence of wormlike structures in both the plain polymer and the BT44 formulation. The BT44 formulation retained biological activity in immortalized cells and in cultured dopamine neurons. The micellar nanoformulation of BT44 exhibited improved absorption (after subcutaneous injection) and blood–brain barrier (BBB) penetration, and no acute toxic effects in mice were observed. In conclusion, herein, we have developed an ultrahigh BT44-loaded aqueous injectable nanoformulation, which can be used to pave the way for its preclinical and clinical development for the management of neurodegenerative disorders.

## Introduction

In the past decades, drug discovery has seen significant advancements in technological innovation, resulting in the development of a large number of potential candidates as drug molecules. However, according to some estimates, 90% of the drugs in the development pipeline and 40% of the drugs in the market are highly hydrophobic (having poor aqueous solubility).^1^ Unfortunately, poor intrinsic aqueous solubility is a common factor in both synthetic and naturally occurring pharmacophores,^2^ posing a significant risk for varied oral absorption and requiring special vehicles for injectable applications. The aqueous solubility plays a pivotal role in the overall product design to attain higher bioavailability and optimum therapeutic concentration at the site of interest.

The aging population brings new challenges and demands to the society in the context of drug development. Elderly people often suffer from comorbidities that require diverse pharmacological management. In particular, currently neurodegenerative disorders such as Parkinson’s disease (PD), retinal degeneration, amyotrophic lateral sclerosis, and neuropathic pain affect an ever-increasing number of patients in modern society. These disorders are caused by the death of various neuronal populations in the body and are currently incurable.^3^ Potential disease-modifying treatments can be developed on the basis of neurotrophic factors and secreted proteins which function as the survival and maintenance factors for both developing and mature neurons.^4^

Glial cell line-derived neurotrophic factor (GDNF) is the most promising protein for the treatment of PD. GDNF first binds to glycosylphosphatidylinositol-anchored coreceptor GDNF family receptor α and then forms a complex with rearranged during transfection (RET) receptor tyrosine kinase, resulting in the activation of RET. This leads to the activation of RET downstream signaling cascades necessary for cellular processes such as migration, proliferation, differentiation, and metabolism.^4^ However, GDNF lacks druglike properties, making its clinical translation more challenging. Previously, we have designed and developed a series of RET agonists that can bind and activate RET receptors similarly to GDNF.^5,6^ Among them, BT44 is a second-generation compound that alleviated neuropathic pain in both surgery-based and diabetes-induced models of neuropathic pain in rat models.^7^ Further, BT44 also protected cultured dopamine neurons from neurotoxic damage. It also alleviated motor symptoms and protected dopaminergic fibers in the striatum of rats with 6-hydroxydopamine (6-OHDA)-induced PD.^8^ Therefore, it is a promising lead compound for the treatment of neurodegenerative disorders and neuropathic pain.^7,8^ However, like many other hydrophobic drugs, BT44 also suffers from intrinsic poor aqueous solubility, posing significant hurdles at various levels for its preclinical development and further clinical translation.

Various methods such as the preparation of salts forms, drug complexes, use of cosolvents,^9^ drug emulsification, micro/nanonization, crystal engineering, solid dispersion,^10^ and nanotechnological approaches are used to enhance the (apparent) aqueous solubility of such molecules. Among all, the nanocarrier approach is a rapidly emerging tool to address such issues.^11^ A plethora of studies showed the utilization of nanoparticles, nanospheres, nanocapsules, nanosuspension/-emulsion, and micelles for drug delivery applications mainly in the context of solubility enhancement.^12^

Besides other technologies, over the last 30 years, polymer micelles (PMs) have evolved as promising hydrophobic drug solubilization and/or delivery vehicles.^13,14^ They are self-assembled colloidal particles made up of block copolymers composed of hydrophilic and hydrophobic domains.^15^ It is commonly believed that the hydrophobic core is responsible for drug encapsulation, and the hydrophilic shell interacts with solvent molecules (representing core–shell morphology) providing colloidal stability.^16^ However, depending upon the nature of the hydrophilic domain, cargo, and employed (cargo) load, it is becoming more evident that at a certain threshold cargo concentration, the hydrophilic domain also starts to interact^17−22^ with the cargo, indicative of much more complex morphologies.^23,24^ To date, many polymeric micellar formulations have undergone preclinical and clinical trials, presenting improved pharmacological activity with lower systemic toxicity, but successful translations into clinics were few.^14,25,26^ Like other excipients used for formulation development, amphiphilic block copolymers used as vehicles for such formulations must also be pharmacologically inert and clinically safe.^27^

In the past decades, polymers of cyclic imino ethers,^28^ particularly poly(2-oxazoline)s (POx) and poly(2-oxazines)s (POzi), have gained significant attention because of their potential in tissue engineering,^29,30^ drug delivery,^26,31−37^ and 3D (bio)printing.^30,38,39^ On the basis of a highly variable molecular toolbox, fine-tuning of the amphiphilic character in AB diblock, ABA triblock (where A and B are hydrophilic and hydrophobic domains, respectively), or more complex architectures^40^ is readily achieved.^41−43^ In particular, ABA triblock copolymers featuring poly(2-methyl-2-oxazoline) (pMeOx) as hydrophilic A block and moderately hydrophobic poly(2-*n*-butyl-2-oxazoline) (pBuOx), poly(2-*n*-butyl-2-oxazine) (pBuOzi), or poly(2-*n*-propyl-2-oxazine) (pPrOzi) showed interesting properties, such as ultrahigh drug loading and excellent cytocompatibility, and appeared to be well tolerated upon *in vivo* administration.^32,38,44^ More importantly, they allow significant therapeutic improvements as already demonstrated for several drugs and drug combinations.^26,45−47^ Accordingly, here we report on a micelle-based nanoformulation of BT44, a compound which has poor intrinsic aqueous solubility, using POx-based ABA triblock copolymers. To the best of our knowledge, this represents the first example of any micellar BT44 nanoformulation with a detailed physicochemical characterization which retains biological activity and demonstrates improved absorption and better blood–brain barrier (BBB) penetration compared to the BT44 dissolved in propylene glycol (PG) upon *in vivo* administration.

## Materials and Methods

All substances for the synthesis of polymers were purchased from Sigma-Aldrich (Steinheim, Germany) or Acros (Geel, Belgium) and were used as received unless otherwise stated. The monomers used in this study are 2-*n*-propyl-2-oxazoline (PrOx), 2-*n*-butyl-2-oxazoline (BuOx), and 2-*n*-pentyl-2-oxazoline (PentOx). The monomer PentOx was particularly synthesized for this study, following a modified procedure of Witte and Seeliger^48^ as reported recently.^41,49^ All the other substances used for polymerization, i.e., methyl trifluoromethylsulfonate (MeOTf) and 2-methyl-2-oxazoline (MeOx), and solvents for polymer synthesis were refluxed over calcium hydride, while benzonitrile (PhCN) was refluxed over P_2_O_5_ and distilled under argon. Deuterated solvents for NMR analysis were purchased from Deutero GmbH (Kastellaun, Germany).

### Monomer Synthesis

One equivalent of hexanenitrile, 1.2 equiv of ethanolamine, and 0.025 equiv of zinc acetate dihydrate were added to a nitrogen-flushed flask and heated to 130 °C (Supporting Information, synthesis 1). The reaction was kept under reflux (for 4 days) until the reaction mixture turned dark brown. The reaction progress was controlled by ^1^H NMR spectroscopy. The raw product was dissolved in chloroform (30 mL) and washed with deionized (DI) water (60 mL, 3 times). The organic phase was collected and dried with sodium sulfate, filtered, and concentrated under vacuum. The residue was mixed with calcium hydride and distilled under vacuum. If necessary, the distillation was repeated, and the product was stored under argon atmosphere.

### Polymer Synthesis

The polymerizations and workup procedures for A-pPrOx-A and A-pBuOx-A triblock copolymers have been previously reported.^41,49−51^ The synthesis of A-pPentOx-A triblock copolymer was performed as follows (for details, see the Supporting Information, synthesis 2). The initiator, MeOTf, was added to a dried and argon-flushed flask followed by the addition of PhCN. Further, the monomer for the first hydrophilic block (A), i.e., MeOx, was added, and the reaction mixture was heated to 110 °C and incubated under continuous stirring for approximately 4 h. The reaction progress was controlled by ^1^H NMR spectroscopy. After complete polymerization of MeOx, the mixture was cooled to room temperature, and the monomer for the second block, i.e., PentOx, was added. The reaction mixture was heated to 110 °C and kept stirring overnight. The procedure was repeated for the third block (MeOx), and after confirmation of full monomer consumption, termination was carried out by the addition of 5 equiv of 1 M sodium hydroxide aqueous solution, and the mixture was stirred at 50 °C for 4 h. The PhCN was removed under reduced pressure. The highly viscous polymeric residues were dissolved in DI water, transferred into a dialysis bag, and dialyzed against DI water for 24 h. The solution was recovered from the bag and lyophilized. The product was obtained as a white powder (yield = 92%).

### Lead Compound: BT44

BT stands for Baltic Technology, a company that was originally involved in the discovery of this scaffold. The chemical name for the BT44 is ((4,5-((3,4-dihydroisoquinolin-2(1*H*)-yl) sulfonyl)-2-methoxyphenyl)piperazin-1-yl (4-fluoro-2 (trifluoromethyl)phenyl)methanone, with a molecular weight of 577.59. The compound was synthesized by EvoBlocks (Hungary, Cat# EBR-10719615). The chemical structure of BT44 was verified by ^1^H NMR spectroscopy. The experiments were performed on a Bruker Avance III HD NMR spectrometer operated at a 1H frequency of 850.4 MHz equipped with a cryogenic probe head by the NMR facility at the Institute of Biotechnology, University of Helsinki, Finland. The purity was further determined by HPLC (97.3%).

### Nuclear Magnetic Resonance Spectroscopy (^1^H NMR)

The ^1^H NMR spectra of the monomers, polymers, and nanoformulations were recorded on a Fourier 300 (300.12 MHz) instrument, Bruker Biospin (Rheinstetten, Germany) at 298 K. The spectra were calibrated to the signal of the residual protonated solvent (CDCl_3_ at 7.26 ppm or D_2_O at 4.79 ppm) and analyzed using MestReNova software (version 6.0.2-5475).

### Dialysis

The synthesized polymer was dialyzed using Spectra/Por membranes with a MWCO of 1 kDa (material: cellulose acetate) obtained from neoLab (Heidelberg, Germany). The DI water was renewed after 1, 4, 12, and 24 h, and the resulting polymer solution was lyophilized for 48 h. After lyophilization, the polymer was obtained as white flakes.

### Gel Permeation Chromatography (GPC)

GPC was performed on a SECurity GPC Agilent 1260 Infinity System (Polymer Standard Service, Mainz, Germany) with HFIP containing 3 g/L potassium trifluoroacetate; precolumn: 50 × 8 mm PSS PFG linear M; 2 columns: 300 × 8 mm PSS PFG linear M (particle size 7 μm; pore size 0.1–1000 kDa). The columns were kept at 40 °C, and the flow rate was 0.7 mL/min. Prior to each measurement, samples were filtered through 0.2 μm Teflon (PTFE) filters, Roth (Karlsruhe, Germany). Conventional calibration was performed using poly(ethylene glycol) standards (0.1–1000 kg/mol), and data was processed with Win-GPC software and further plotted in OriginPro 2015 Sr2 (version b9.2.272) software.

### Thermogravimetric Analysis (TGA)

TGA analysis was performed on a TG 209 F1 IRIS instrument, NETZSCH (Selb, Germany). The pristine polymer sample as a powder (10–15 mg) was placed in aluminum oxide crucibles (NETZSCH, Selb, Germany) and heated from 30 to 900 °C with a heating rate of 10 K/min under synthetic air while detecting the mass loss.

### Polymer–Drug Compatibility by Solubility Parameters

The extent of compatibility between BT44 and the hydrophobic block of A-B-A triblock copolymers was estimated by using the Hildebrand–Scatchard equation.^52^
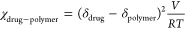
1where χ_drug–polymer_(χ_dp_) represents the Flory–Huggins interaction parameter, and δ_drug_ and δ_polymer_ are the solubility parameters for the lead compound BT44 and polymer, respectively. The molar volume *V* of BT44 was calculated by Fedor’s method,^53^ and *R* and *T* are the gas constant and the temperature in K, respectively. δ_drug_ and δ_polymer_ were calculated by following equations:
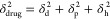
2
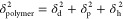
3δ_drug_ and δ_polymer_ represent the total solubility parameters (δ_total_) for the drug and polymer, respectively. δ_total_ is the sum of the dispersion (δ_d_), polar (δ_p_), and hydrogen-bonding contributions (δ_h_). δ_d_, δ_p_, and δ_h_ were further calculated by Hoftyzer and Van Krevelen’s additive group contribution method by using the following equations:
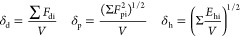
4where *F*_di_, *F*_pi_, and *E*_hi_ are the molar dispersion, polar attraction constant, and hydrogen-bonding energy, respectively. Each structural group in the molecule contributes toward *F*_di_*, F*_pi_, and *E*_hi_, and the subsequent values were obtained from the literature.^54^

### Drug Formulation

The BT44-loaded micellar formulations were prepared by using the thin-film hydration method. Initially, the polymer (10 or 100 g/L) and the BT44 (15 g/L) stock solutions in ethanol (EtOH) were prepared individually followed by mixing in the desired ratios in Eppendorf tubes. Under a mild stream of argon, the EtOH was removed by placing the Eppendorf tubes in a water bath at 50 °C, resulting in thin films. To remove the traces of EtOH (if any), the films were further dried *in vacuo* (≤0.2 mbar) for at least 30 min. Subsequently, preheated DI water (37 °C) was added to obtain the desired final polymer (10 or 100 g/L) and BT44 concentrations followed by shaking at 55 °C for 15–30 min at 1250 rpm with a Thermomixer comfort (Eppendorf AG, Hamburg, Germany) to ensure complete solubilization. The prepared BT44 formulations were subjected to centrifugation for 5 min at 10 000 rpm with a 3-Speed microcentrifuge, (neoLab, Heidelberg, Germany) to remove (or sediment) nonsolubilized BT44 (if any). The formulation experiments were performed in triplicates, and results are presented as mean ± standard deviation (SD).

### High-Performance Liquid Chromatography (HPLC)

The quantification of BT44 was performed by HPLC. The details of the HPLC setup are as follows: an LC-20A Prominence HPLC (Shimadzu, Duisburg, Germany) equipped with a system controller CBM-20A, a solvent delivery unit LC-20 AT (double plunger), an online degassing unit DGU-20A, and an autosampler SIL-20AC. The detector is a photodiode array detector SPD-M20A. A ZORBAX Eclipse Plus (Agilent, Santa Clara, CA) C18 column (4.6 × 100 mm; 3.5 μm) was used as the stationary phase. Each HPLC measurement took 18 min with a flow rate of 1 mL/min. The quantification was performed with a stepwise gradient using acetonitrile (ACN) and water with 0.05% TFA. Within the first 10 min, the ratio of H_2_O/ACN was gradually changed from 60/40 (v/v) to 40/60 (v/v). Within the next 10 s, the solvent was abruptly changed to 20/80 (v/v) and kept constant for 2 min, followed by a sudden change back to 60/40 (v/v). This ratio was kept constant for the next 8 min followed by the start of the second run. The detection was performed at 230 nm, and the retention time of BT44 was found to be 12.6 min.

### Loading Capacity and Loading Efficiency

The following equations were used to calculate the loading capacity (LC) and loading efficiency (LE) of the BT44 formulations:
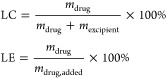
where *m*_drug_ and *m*_excipient_ are the weight amounts of the solubilized drug and polymer excipient in solution and *m*_drug,added_ is the weight amount of the drug initially added to the dispersion. No loss of polymer during micelles preparation was assumed.

### Stability Studies

For stability studies, all the freshly prepared BT44 formulations were stored at ambient conditions (≈ 25 °C) under the exclusion of light. Before the determination of the drug loading by HPLC, all samples were centrifuged for 5 min at 10 000 rpm with a 3-Speed micro centrifuge (neoLab, Heidelberg, Germany) to sediment any aggregates or agglomerates. The samples from the supernatant were taken at day 0, 1, 5, and 15 followed by quantification with HPLC (as described in the HPLC section). All the formulations and stability experiments were performed with three individually prepared samples, and results are presented as means ± SD.

### Redispersion Studies

The freshly prepared BT44 formulations were frozen in liquid nitrogen and subjected to 24 h lyophilization to get the BT44 formulation as a dried powder for redispersion studies. The lyophilized formulations were redispersed in normal saline (0.9% NaCl) and cell culture media (Dulbecco’s modified eagle medium, DMEM). After the addition of the solvent of interest, the formulations were shaken at 1250 rpm with a Thermomixer comfort (Eppendorf AG, Hamburg, Germany) at room temperature for 5 min followed by HPLC analysis as stated in the HPLC section.

### Differential Scanning Calorimetry (DSC)

DSC was performed on a DSC 204 F1 Phoenix equipped with a CC200 F1 Controller (NETZSCH, Selb, Germany). The dynamic scans were recorded in a nitrogen atmosphere with a heating rate of 10 K/min (0–200 °C). For DSC studies, samples were placed into flat-bottom aluminum pans with pierced lids. Prior to DSC measurements, the aqueous BT44 formulations were lyophilized to obtain the dry powder. The DSC of plain polymer and pristine BT44 was also performed. The data was further analyzed in thermoanalysis software and plotted in OriginPro 2015 Sr2 (version b9.2.272) software.

### X-ray Diffraction (XRD)

Powder XRD of the lyophilized BT44 formulation was performed on X-ray diffractometer D8 Davinci Design (Bruker AXS). The setup was equipped with a radiation source of Cu–Kα, and the detector was a linear position sensitive detector (PSD). Measurement were done at 0.02° steps, each step lasting 1 s in transmission mode at 40 kV voltage, and 40 mA current and 2Θ angle ranging from 5° to 40° were used. The XRD of plain polymer and pristine BT44 was also performed under the same set of conditions. The data was further analyzed and plotted in OriginPro 2015 Sr2 (version b9.2.272) software.

### Dynamic Light Scattering (DLS)

The DLS was performed using a Zetasizer Nano ZSP from Malvern (at a single angle of 173°) (Malvern Instruments, Worcestershire, U.K.) in disposable cuvettes (UV cuvettes semi micro, BRAND GmbH, Wertheim, Germany) at ambient temperature (≈25 °C). The plain polymer and various BT44 formulations were measured after filtration through a 0.45 μm PVDF syringe filter (Rotilabo, Karlsruhe, Germany). The measurements were recorded as the average of three test runs (each with 12 submeasurements) for one individually prepared sample. Data was analyzed by using zetasizer software 7.11 and plotted in OriginPro 2015 Sr2 (version b9.2.272) software.

### Cryogenic Transmission Electron Microscopy (cryo-TEM)

The freshly prepared plain polymer solutions with concentrations of 10 and 25 g/L (as free-flowing liquid solution) and the redispersed polymer/BT44 formulation at 10/2 g/L feed (*n* = 1) were subjected to cryo-TEM visualization. For sample preparation, copper grids coated with holey carbon support film (quantifoil, 400 mesh, R1/2) were glow-discharged in air for 1.5 min in a plasma cleaner (Harrick PDC-002). Afterward, 5 μL of each sample solution was applied on the grids and plunge-frozen in liquid ethane with a Vitrobot IV (FEI Company, Hillsboro, OR). The humidity in the chamber was set to 100%, and the temperature was kept at 25 °C. The following settings were further employed: wait time 0 s, drain time 0 s, blot time 5 s, and blot force −5. The samples were imaged on an FEI Tecnai T12 Spirit transmission electron microscope (FEI Company, Hillsboro, OR) equipped with a LaB6 emitter at an acceleration voltage of 120 kV and a temperature of −180 °C. The images were recorded with an Eagle CCD camera (FEI Company, Hillsboro, OR) in low-dose mode with a total dose of 30 e/Å^2^ and a nominal underfocus of −3 μm with serial EM.^55^ The images were further processed in ImageJ software (1.46 r, revised edition).

### Cryogenic Scanning Electron Microscopy (cryo-SEM)

In comparison to cryo-TEM, relatively much higher plain polymer concentrations, i.e., 50 and 100 g/L (as free-flowing liquid solution) were visualized with cryo-SEM. The samples were rapidly frozen in slushed nitrogen at −210 °C after being placed between aluminum plates (*d* = 3 mm) with a 2 mm notch for sample fixation. All the following transfer steps were performed at −140 °C with an EM VCT100 cryo-shuttle (Leica Microsystems). To generate a freshly fractured polymer surface, one of the aluminum plates was knocked off and freeze etched for 15 min at −85 °C under high vacuum (<1 × 10^3^ mbar) in a Sputter Coater machine (ACE 400, Leica Microsystems). Afterward, samples were sputtered with 3 nm of platinum and transferred to the SEM chamber (Crossbeam 340, Zeiss). The images of the polymer surface morphology were taken at −140 °C using an acceleration voltage of 8 kV. The images were further processed in ImageJ software (1.46 r, revised edition).

### *In Vitro* Studies

#### Cytocompatibility Study of the Polymer Excipient

The cytocompatibility study of the plain polymer (A-pPentOx-A) solution was carried out on MG87 RET cell lines. The cell viability was examined using Alamar blue assay as described previously.^56^ The MG87 RET cells were cultured overnight in 96-well plates (OptiPlate 96 F HB, Wallac). Various concentrations of polymer, i.e., 1, 5, 10, and 50 g/L, dissolved in DMEM and 15 mM HEPES were applied to the cultured cells. At 72 h post-treatment, Alamar blue was dissolved in the cell culture media in the ratio 1:10 and applied to the cultured cells. After 2 h of incubation with Alamar blue reagent, the fluorescence was measured using a Victor plate reader at an excitation and emission wavelength of 540 and 590 nm, respectively. The experiments were repeated three times independently.

#### Luciferase Assay

Luciferase assay was performed as described earlier.^5,6^ The cells (reporter cell line) were plated in 96-well cell culture plates in 100 μL of media (15 mM HEPES pH 7.2, 1% DMSO, 100 of μg/mL normocin). The next day, the cells were exposed to several concentrations of pristine BT44 and BT44 formulation (1, 5, 10, 25, 50, and 100 μM) in DMEM, 15 mM HEPES, pH 7.2, and 1% DMSO-containing media. The concentrations were prepared 2× that of the desired final concentration, and 100 μL of the treatment was added to the cells and incubated overnight. The next day, the condition of the cells was visually assessed (to evaluate potential toxic effects of the treatments). According to the manufacturer’s instruction, the luciferase activity was measured using neolite reagent (PerkinElmer, USA). The luminescence was detected using a Victor plate reader. The experiments were repeated three times independently.

#### RET Phosphorylation Assay

RET phosphorylation assay in response to BT44 was carried out in MG87 RET cells transfected with hGFRα1 plasmids as described previously.^6^ In short, MG87 RET cells were cultured in 6-well plates in DMEM with 10% FBS. One day prior to the experiment, 100 μg/mL normocin was added into each well. Subsequently, the cells were transfected with 4 μg/well of GFRα1-expressing plasmid using Lipofectamine 2000 (Invitrogen) as described by the manufacturer. The cells were starved (starvation media: serum-free DMEM, 15 mM HEPES, pH 7.2) for 4 h and stimulated for 15 min with 0.64 mg/mL (similar to 79 μM) of plain polymer, 100 μM BT44 nanoformulation, 100 μM pristine BT44, or 6.6 nM GDNF (PeproTech, Ltd.) dissolved in starvation media. After 15 min, the cells were washed once with ice-cold PBS containing 1 mM Na_3_VO_4_ and lysed with RIPA-modified buffer (50 mM Tris-HCl, pH 7.4, 150 mM NaCl, 1 mM EDTA, 1% NP-40, 1% TX-100, 10% glycerol, EDTA-free protease inhibitor cocktail (Roche, Switzerland), 1 mM Na_3_VO_4_, 6 mM sodium deoxycholate, and 1 mM PMSF) on ice followed by centrifugation. The supernatant obtained was used to immunoprecipitate RET with RET antibody (2 μg/mL, R&D, Cat# AF1485) bound to magnetic beads coated with protein G (Dynabeads Protein G, Life Technologies, USA). The precipitated immunocomplexes were resolved on SDS–PAGE, transferred to nitrocellulose membranes, and probed with antiphosphotyrosine antibodies (1:1500, clone 4G10, Merck Millipore, Germany, Cat# 05-321). The stained bands were visualized with enhanced chemiluminescence (ECL) reagent (Pierce) using LAS3000 imaging software. To ensure equal loading, membranes were stripped and restained with anti-RET C-20 antibody (1:500, R&D, Cat# AF1485).

The images obtained from the Western blot were quantified using Image Studio 5.2 software (LI-COR Biosciences, USA), and the band intensities of phosphorylated form of RET were normalized to RET. The quantification was done for images from two independent experiments.

#### Cell-Based ^125^I-GDNF-Displacement Assay

The cell-based ^125^I-GDNF-displacement assay was carried in HEK293 cells. The cells were cultured in DMEM, 10% FBS, and 100 μg/mL of normocin overnight and then transfected with GFRα1 or GFRα1 and RET using Lipofectamine 2000 (Invitrogen), as described by the manufacturer. The next day, the cells were incubated for 1 h on ice in the presence of the BT44 formulation in concentrations ranging from 0 to 50 pM. Afterward, 50 pM of iodinated GDNF was added, and the cells were incubated for 1 h on ice with ^125^I-GDNF and nanoformulated BT44. Subsequently, cells were washed with PBS four times and lysed with 1 M NaOH, and lysates were collected into the scintillation vials and counted using a PerkinElmer Wallac Wizard 1470-020 Gamma Counter. The data for GFRα1-transfected cells was collected in two independent experiments and for GFRα1/RET-transfected cells in four independent experiments. The binding data were analyzed by nonlinear regression analysis using GraphPad Prism 8.4.2 software to determine the IC_50_ values. Experiments with nonformulated BT44 were not conducted because of insufficient solubility of the compound in assay media.

#### Binding Assay Using Microscale Thermophoresis

The molecular interaction between nanoformulated BT44 and the receptors was studied using microscale thermophoresis (MST). Nonformulated BT44 was not studied in this assay owing to insufficient solubility. All the experiments were performed using a Monolith NT.115 instrument (NanoTemper Technologies GmbH, Germany). Recombinant human GFRα1 or RET extracellular domain was labeled through His-tag using the Monolith His-Tag Labeling Kit RED-tris-NTA (NanoTemper Technologies GmbH; MO-L008). The His-labeled GFRα1 or RET was used at a 20 nM concentration. The starting concentration of nanoformulated BT44 (ligand) was 10 μM, which was chosen based on the result obtained from cell-based experiments. All the measurements were performed using premium-coated capillaries (NanoTemper Technologies GmbH; MO-K025) in a buffer containing 20 mM HEPES, 150 mM NaCl, 186 μM CaCl_2_, and 0.05% Tween-20. The measurements of the interaction between the formulation and the receptors were carried using a red LED source, power set at 100%, and medium MST power at 25 °C. The data were obtained from three independent experiments and were analyzed using MO. The affinity analysis software v2.3 was used for analysis, and the dissociation constant (*K*_d_) was further calculated.

#### Neuroprotective Effect of Nanoformulated BT44 in Primary Dopamine Neurons Culture

The neuroprotective effect of the plain polymer, nanoformulated BT44, and pristine BT44 (dissolved in dimethyl sulfoxide (DMSO)) was studied in 1-methyl-4-phenylpyridinium (MPP+)-challenged dopamine neurons as described previously.^5,8,56^ The midbrain dopamine neurons were isolated from E13.5 embryos of NMRI mice and cultured for 5 days in dopamine neuron culture medium [Dulbecco’s MEM/Nut mix F12 (Invitrogen/Gibco, Cat# 21331-020), 1 × N_2_ serum supplement (Invitrogen/Gibco, Cat# 17502-048), 33 mM d-glucose (Sigma-Aldrich, Germany, Cat# G-8769), 0.5 mM l-glutamine (Invitrogen/Gibco, Cat# 25030-032), and 100 μg/mL Primocin (InvivoGen, USA, Cat# ant-pm-2)]. On day 6, plain polymer (i.e., 0.0064 mg/mL similar to 790 nM), nanoformulated BT44 (100 nM), or DMSO BT44 (100 nM) and GDNF (3.3 nM) were added to the cultured cells for a period of 48 h. Afterward, the media were removed, and the cells were fixed with 4% paraformaldehyde (PFA) for 20 min, washed with PBS, and permeabilized with 0.2% TritonX-100 in PBS. Further, the cells were blocked with 5% horse serum for 1 h and then incubated with mouse anti-TH antibody (1:2000, Merck Millipore, Cat# MAB318) overnight at 4 °C. Subsequently, cells were washed and incubated with Alexa Fluor 647 conjugated donkey antimouse secondary antibody (1:500, Thermo Fisher Scientific, USA, Cat# A-31571) and 0.2 μg/mL of DAPI (4′,6-diamidino-2-phenylindole) for 1 h at room temperature. Finally, cells were imaged by an ImageXpress Nano Automated Imaging System (Molecular Devices) at 10× magnification. The images were analyzed using CellProfiler image analysis software to calculate the number of tyrosine hydroxylase (TH) positive cells. The resulting data were subjected to statistical analysis in GraphPad Prism software.

### *In Vivo* Studies

#### Experimental Animals

The test animals, i.e., mice used in the experiments were housed under 12 h light–dark cycles with food and water available ad libitum. The experiments were carried out in accordance with 3R principle, European Community guidelines for the use of experimental animals and approved by the National Animal Experiment Board of Finland (license numbers ESAVI/7551/04.10.07/2013 and ESAVI/198/04.10.07/2014) for experiments with living animals. Animals for tolerability studies were leftover animals from breeding experiments (mouse line: C57BL/6JRccHsd and B6C3-Tg (HD82Gln) 81Gschi/J). Both male and female mice (age = 4–5 months) were used for the experiments. The use of animals for the primary neuronal culture was approved by the Laboratory Animal Centre of the University of Helsinki (license number KEK15-022). The primary dopamine culture was prepared from E13.5 embryos of NMR1 mice.

#### *In Vivo* Assessment of the Tolerability of BT44 in Propylene Glycol or as an Aqueous POx Formulation

The BT44 tolerability in mice was studied in experiments with crossover design. The mice (*n* = 24) were randomly divided into eight groups (three mice in each group). In the first period of the experiment, mice from group one to eight were subcutaneously injected with normal saline, propylene glycol (PG), plain polymer (355 and 586 mg/kg), nanoformulated BT44 at 25 and 50 mg/kg, and BT44 dissolved in PG (25 and 50 mg/kg), respectively. The subcutaneous injection volume was 10 mL/kg. The amount of plain polymer used was the same as the polymer present in the nanoformulated BT44 in the *in vivo* experiment. After a 7 day washout period, study agents were reshuffled between the groups so that each group received a different study agent after each washout period. One hour after injection, mice were placed in the corner of an Open-field chamber (30 × 30 cm, Med Associates), and the locomotor activity was monitored for 15 min. The health condition of the mice was monitored by experienced personnel on a daily basis during the entire time span of the experiment. After the second treatment period, animals were terminated, and blood and brain were collected for absorption and BBB penetration analysis.

#### Determination of the BT44 Concentration in Blood and Brain

Both blood and brain concentrations of BT44 were determined 1 h after single subcutaneous injection of 50 mg/kg of nanoformulated BT44 or BT44 dissolved in PG in mice (*n* = 4–6 mice per group). One hour post subcutaneous injection, mice were anaesthetized using terminal phenobarbital injection (50 mg/kg), and blood samples (0.3–0.8 mL) were collected by cardiac puncture with surgical heart exposure (open method) followed by storage in EDTA tubes (Microvette 100 K3E, Sarstedt). The plasma fraction was separated by centrifugation (3000 rpm, 5 min, 4 °C) and placed on dry ice followed by storage at −72 °C until analysis. For the measurement of the brain concentration of BT44, mice were transcardially perfused with an ice-cold normal saline, and the brain tissues were collected and snap frozen in liquid nitrogen. The concentration of BT44 in both the plasma and brain was measured by UPLC coupled with time-of-flight mass spectrometry. The measurements were performed by Pharmidex Pharmaceutical Services Ltd. (U.K., https://www.pharmidex.com/).

#### Statistical Analysis

The statistical analysis was done using the Student’s *t* test or one-way ANOVA with a Dunnett’s or Tukey HSD *post hoc* test in GraphPad Prism 8 (GraphPad Software Inc., USA). The differences with *p* values below 0.5 were considered statistically significant.

## Results and Discussion

### Polymer Synthesis and Characterization

POx-based ABA triblock copolymers have shown great potential to deal with difficult to solubilize drugs.^26,33,35,57^ In the majority of cases, the hydrophilic block A is pMeOx, while the hydrophobic block B can be from a series of linear,^58,59^ branched (aliphatic POx with varying side chain lengths; C4–C9),^41,51^ or aromatic ring side chains.^34,50^ For POx-based amphiphiles, it has been established repeatedly that a minimal contrast in hydrophilic/lipophilic domains is beneficial^51^ for high drug loading.^33,34,46,49,50,60^ Among many POx-based triblock copolymers,^41^ the most repeatedly explored amphiphile is A-pBuOx-A^34,35,43^ allowing, *inter alia*, ultrahigh drug loading for paclitaxel (PTX) (≈50 wt %).

**Figure 1:**
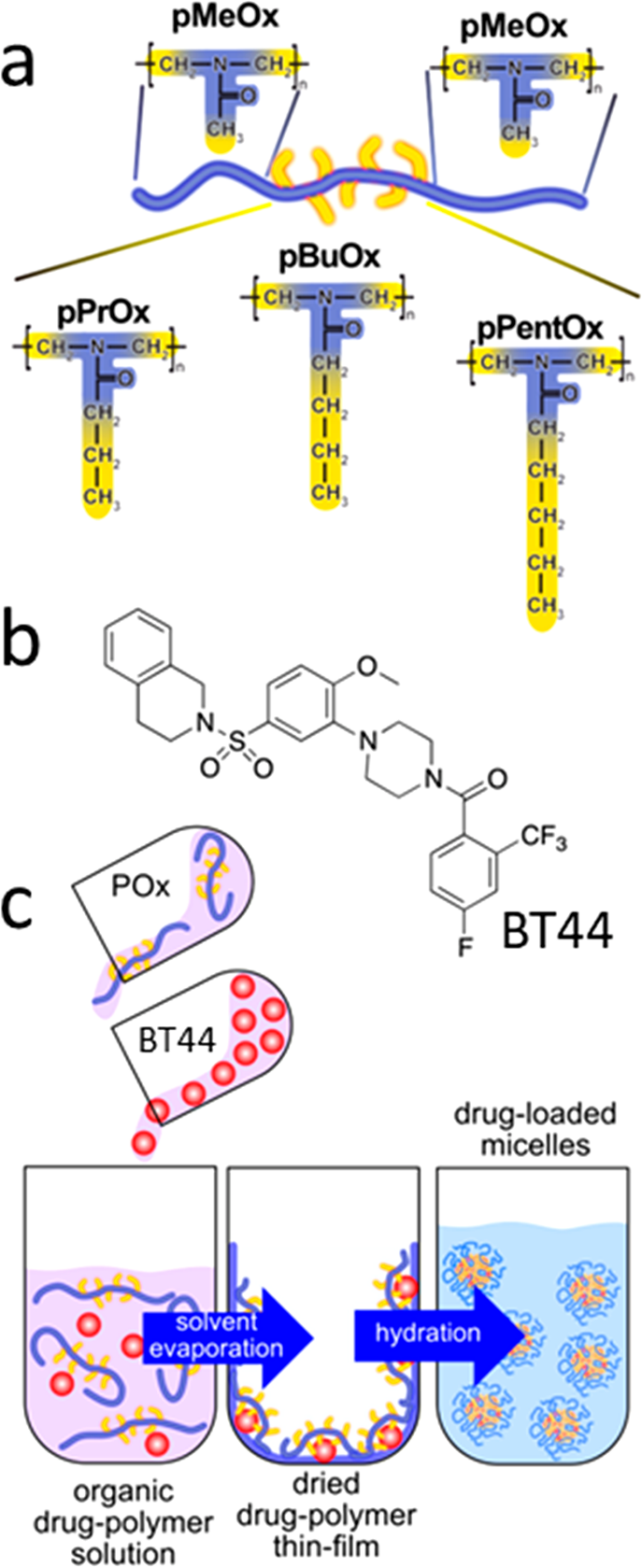
(a) Schematic representation of the triblock copolymers used in this study representing hydrophilic and hydrophobic domains. (b) Chemical structure of BT44. (c) Schematic illustration of the thin-film hydration method used for the formulation development.

Taking this into account, we selected three POx-based copolymers (including the thoroughly investigated pBuOx-based amphiphile)^26,32,34,35,46^ consisting of a moderately hydrophobic block B, i.e., poly(2-*n*-propyl-2-oxazoline) (pPrOx), pBuOx, and poly(2-*n*-pentyl-2-oxazoline) (pPentOx) (Figure 1a), and hydrophilic pMeOx A blocks. All polymer amphiphiles in this study were synthesized by living cationic ring-opening polymerization (LCROP) as previously described.^49^ The BT44-loaded (Figure 1b) POx-based micellar formulations were prepared using the thin-film hydration method (Figure 1c).

**Figure 2:**
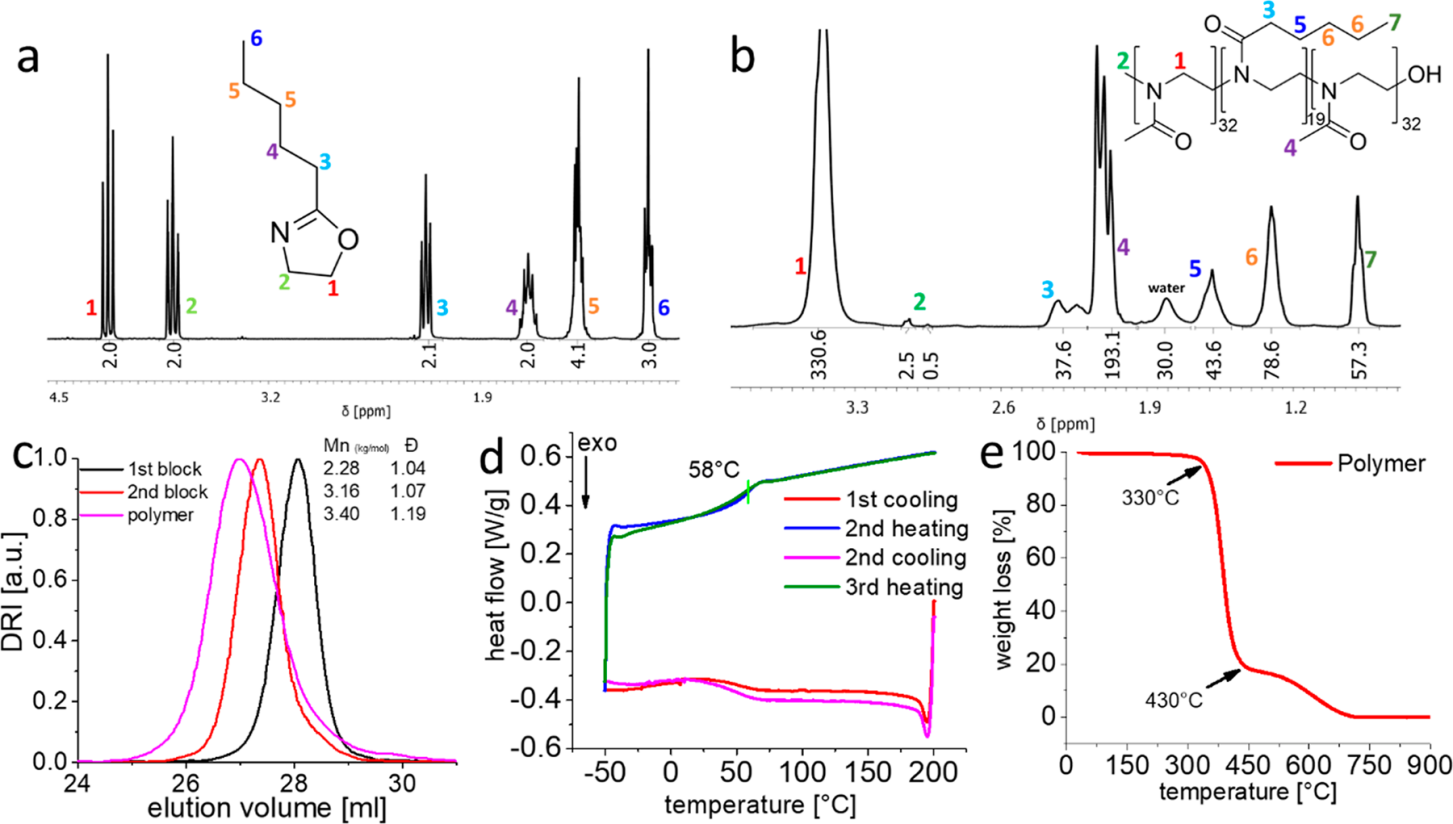
^1^H NMR spectra (CDCl_3_; 300 MHz; 298 K) and chemical structure of (a) 2-*n*-pentyl-2-oxazoline monomer and (b) pMeOx-*b*-pPentOx-*b*-pMeOx triblock copolymer with signal assignment of all major peaks. (c) GPC elugrams (solvent; HFIP, system calibrated with PEG standards) after each polymerized block and purified A-pPentOx-A polymer. (d) DSC thermogram of plain A-pPentOx-A triblock with heat flow occurring during the various heating and cooling cycles (10 K/min); green vertical line indicates the glass transition point at 58 °C. (e) Weight loss occurring during the thermogravimetric analysis of A-pPentOx-A. Samples were heated from 30 to 900 °C at the heating rate of 10 K/min.

As all ABA triblock copolymers are composed of pMeOx as A, they are represented according to the hydrophobic block, i.e., A-pPrOx-A, A-pBuOx-A, and A-pPentOx-A, in the following. The targeted block length for each block in the triblock copolymer is A_35_-B_20_-A_35_. The former two polymers were previously investigated for drug formulation,^41,49,58^ while the A-pPentOx-A triblock copolymer was specifically synthesized for this contribution. Initially, we synthesized the PentOx monomer followed by characterization with ^1^H NMR spectroscopy (see the Supporting Information, synthesis 1). All the signals were unambiguously assigned. The signals of two methylene groups (signal 1 and 2) originating from 2-oxazoline (at 4.2 and 3.8 ppm) confirmed the successful ring formation (Figure 2a). After purification by distillation, the PentOx monomer was polymerized with MeOx (A) to obtain the desired A-pPentOx-A (see the Supporting Information, synthesis 2). The polymerization was terminated with aqueous sodium hydroxide solution. Previously, we had observed relatively little effect of the polymer termini in POx/POzi-based A-B-A triblock copolymers, but this should be assessed on a case-by-case basis.^58,61^ The resulting A-pPentOx-A triblock copolymer was characterized by ^1^H NMR, GPC, DSC, and TGA. The ^1^H NMR spectroscopy showed good synthetic control and agreement to the targeted length for the individual blocks (Figure 2b). During synthesis, after full monomer consumption for the individual block, a small volume of the reaction mixture was collected and analyzed by GPC. With the polymerization of each individual block, a distinct shift to higher molar mass was observed, and the elugrams for the intermediates and final polymer appeared essentially monomodal with reasonably low dispersity (D̵ < 1.20) (Figure 2c). For the synthesis and characterization of A-pPrOx-A and A-pBuOx-A triblock copolymers, the readers are referred to previous reports.^41,49^ To understand the thermal characteristics of the novel polymer, differential scanning calorimetry (DSC) measurements were performed. Corroborating results from previously reported POx-based triblock copolymers,^41^ all the three triblock copolymer amphiphiles exhibited a single glass transition (*T*_g_) suggesting an amorphous sample and the absence of (micro)phase separation in the solid state. As expected, a slight decrease in *T*_g_ was observed with the increasing side chain length in the hydrophobic block, i.e., 65, 63, and 58 °C for A-pPrOx-A, A-pBuOx-A, and A-pPentOx-A, respectively (Figure 2d and Figure S1). The thermogravimetric analysis revealed that A-pPentOx-A was rather stable, and the onset of the first major loss was observed at approximately 330 °C (Figure 2e). This high thermal stability, well-known for POx and POzi triblock copolymers, could be very beneficial for melt processing, e.g., preparation of hot melt extrudates of A-pPentOx-A and drugs of interest.

### Determination of Solubility Parameters

**Figure 3:**
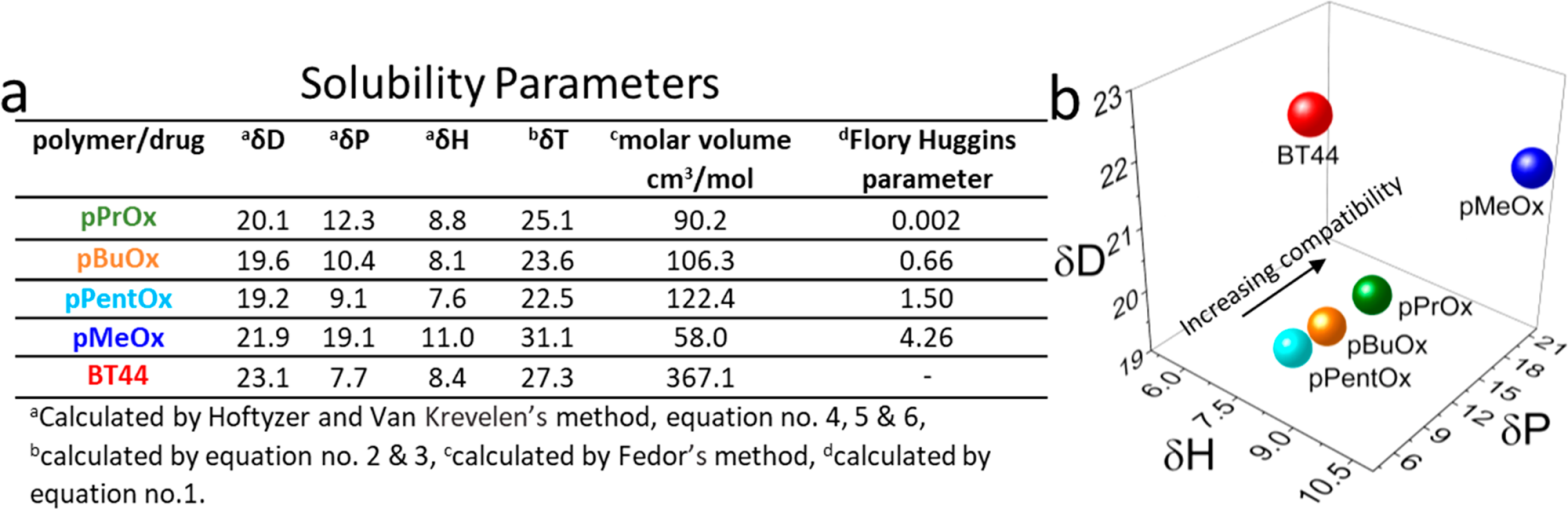
(a) Hansen solubility parameters, molar volume, and Flory–Huggins parameters of BT44 and various blocks of the ABA triblock copolymers. (b) Distribution of solubility parameters for BT44 and various blocks of ABA triblock copolymer in three-dimensional Hansen space.

The physicochemical compatibility between drug and polymer obviously plays a pivotal role in the ideal characteristics of the formulations. The chemical structure of the drug and the polymer (backbone or side chain) can significantly impact the drug loading and stability of the formulation.^49^ Despite some attempts to “predict” drug loading,^37,41,62,63^ drug formulations are mostly still developed by trial and error. Considering the magnitude of the problems of hydrophobic drugs in pharmaceutical technology, finding of a universal carrier/solubilizer seems impossible.^62^ There are a variety of techniques available to estimate or assess the polymer–drug compatibilities^52,64^ in a time- and cost-effective manner. Here, we estimated the compatibility between BT44 and the hydrophobic block in the ABA triblock copolymers theoretically by calculating Hansen solubility parameters (HSPs).^62,65,66^ The HSPs values, i.e., dispersion (δ_d_), polar (δ_p_), and hydrogen-bonding (δ_h_) forces, were calculated using the group contribution method, and the molar volume was calculated by Fedor’s method (Figure 3a and Table S1).^53^ On the basis of the principle of “like dissolves like”, the distance between two materials in the three-dimensional (3D) space is supposed to give an estimate of the compatibility between two substances; therefore, the obtained HSPs values are depicted in 3D space (Figure 3b). Based on the δ_d_, δ_p_, δ_h_, and δ_total_ values, the Flory–Huggin’s interaction parameters (χ_dp_) can be calculated, which is another measure of the compatibility between the polymer and drug wherein a lower value of χ_dp_ (ideally = 0) suggests a higher polymer–drug compatibility. On the basis of the calculated χ_dp_, BT44 compatibility with the three different hydrophobic blocks should be in the order pPrOx > pBuOx > pPentOx, indicating an inverse relationship of side chain length and polymer–drug compatibility. Specifically, pPrOx is suggested to be highly compatible (χ_dp_ ≈ 0.002) and therefore could be assumed to be the best solubilizer for BT44, while pBuOx and pPentOx gave χ_dp_ values of 0.66 and 1.50, respectively, indicative of relatively lower compatibility. The χ_dp_ value for highly hydrophilic pMeOx was ≈4.26, indicating that plain pMeOx homopolymer is thermodynamically a poor solubilizer for BT44. However, this theoretical prediction represents an oversimplification of the system, and the experimental solubilization of BT44 using these three amphiphiles for BT44 must be tested.

### Formulation Studies

Previously, we had observed that a minor change such as shifting of one methylene unit from the side chain to the polymer backbone (within the hydrophobic block) and differences in the side chain have significant impact on the loading capacity for hydrophobic drugs indicative of strong polymer/drug specificities.^41,49^ Here, only a set of three hydrophobic blocks with increasing side chain length, i.e., C3, C4, and C5, were selected and compared for drug-loading capacities.

Accordingly, the solubilization of BT44 was tested with the three triblock copolymers with minimal to moderate amphiphilic contrast (difference of hydrophobicity in the hydrophilic and hydrophobic blocks of the triblock copolymer). Briefly, the polymer and BT44 ethanolic solutions were mixed in the desired ratios (in all cases, the target polymer concentration was kept constant at 10 g/L, while the BT44 concentration was increased from 2 to 10 g/L, unless otherwise stated) followed by ethanol removal and subsequent hydration of the resultant thin film by deionized (DI) water. The (dissolved) BT44 in the micellar formulation was quantified by HPLC (Figure S2) after removal of (if any) nonsolubilized drug particles by centrifugation.

**Figure 4:**
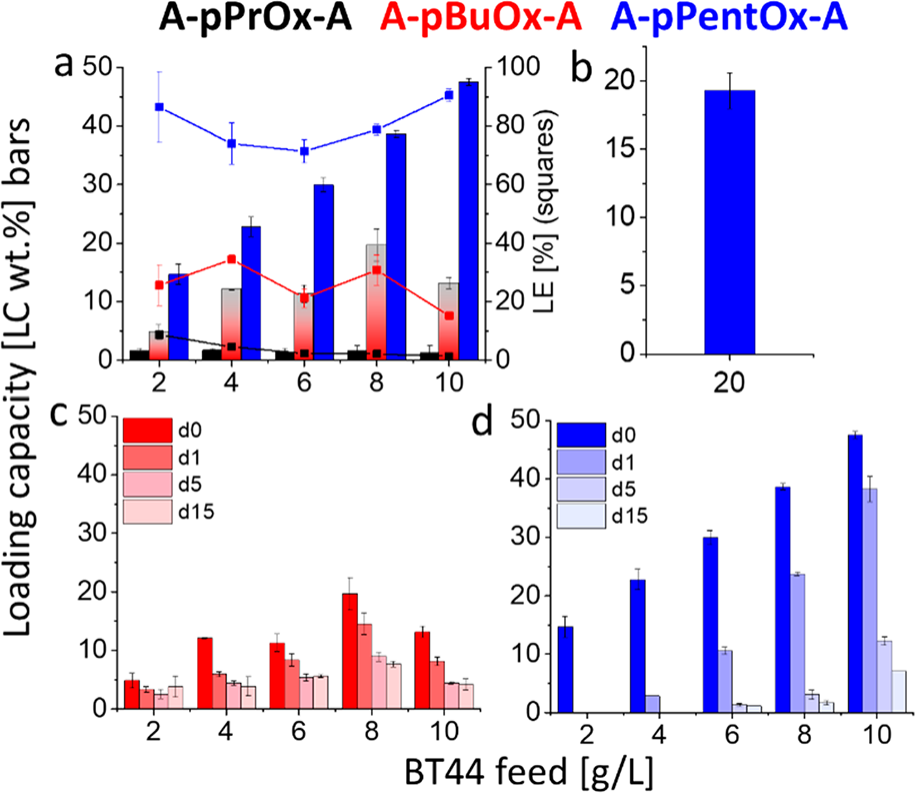
Maximum loading capacity (LC, bars) and loading efficiency (LE, squares) obtained with (a) A-pPrOx-A (black), A-pBuOx-A (red), and A-pPentOx-A (blue) at constant polymer feed of 10 g/L, with increasing BT44 feed from 2 to 10 g/L and with (b) A-pPentOx-A/BT44 feed of 100/20 g/L. Stability study of the (c) A-pBuOx-A and (d) A-pPentOx-A based BT44 formulation stored at ambient conditions (day 0–15) with constant polymer feed of 10 g/L. Data are given as means ± SD (*n* = 3).

Instead of A-pPrOx-A as predicted by solubility parameters or A-pBuOx-A, proven to be the best solubilizer for many drugs,^26,35,43,46^ unexpectedly, A-pPentOx-A gave the highest LC for BT44 (LC ≈ 47 wt %). Increasing the BT44 feed from 2 to 10 g/L raised the LC from 14 to 47 wt % (1.7 to 9.0 g/L) (Figure 4a, blue bars). At the highest LC of 47 wt %, the BT44 formulation appeared as a clear solution with low viscosity (Figure S3a). The LE at all the tested ratios of BT44 ranged between 80 and 90% (Figure 4a, blue squares). For *in vitro* biological activity (or cytotoxicity) studies, relatively low drug concentrations are required (typically nano- to micromolar range), but when it comes to *in vivo* studies, typically a high dose is required owing to the incomplete absorption and (often) large volume of distribution (e.g., the volume of distribution of BT44 is 4.6 L/kg^8^). In addition, ultrahigh drug-loaded formulations are favorable, particularly for injectable administration, because of limitations of the injectable volume, particularly in mouse models. However, one cannot *a priori* assume a high LC also benefits therapeutic outcomes, and some *in vitro* work by Stenzel et al. suggests lower endocytosis of highly drug-loaded polymer micelles,^22^ albeit in certain cases *in vivo* experiments clearly suggest a benefit for highly drug-loaded polymer micelles.^45^ Previously, Lübtow et al. observed that increasing the polymer feed from 10 to 50 g/L aided to increase the aqueous solubility of curcumin (CUR) from 11 to 55 g/L, respectively.^33^ Following this, an additional formulation with higher A-pPentOx-A/BT44 feed, i.e., 100/20 g/L, was prepared. This formulation provided a BT44 aqueous solubility of around 19.3 g/L. However, because of the high polymer feed, the resulting LC was only 16 wt %, albeit with a high LE of 96% (Figure 4b).

In the case of A-pBuOx-A triblock (at 10 g/L polymer feed), the highest LC achieved was 19 wt % (≈2.4 g/L at 10/8 g/L feed) (Figure 4a, red bars). Increasing the BT44 feed did not significantly improve the LC, i.e., falling in the range of 4–19 wt % at all the tested ratios. As a consequence, LE kept on decreasing with the increasing BT44 feed, i.e., 35 to 15% (Figure 4a, red squares). The A-pPrOx-A triblock appeared to be a poor solubilizer for BT44 (LC ≈ 0.5 wt %); at all the tested ratios (Figure 4a, black bars), the flakes of undissolved A-pPrOx-A/BT44 films were clearly visible with the naked eye (Figure S3b) during formulation experiments. In summary, formulation results showed a clear decrease in LC in the order of A-pPentOx-A > A-pBuOx-A > A-pPrOx-A with loading capacities of 47, 19, and 0.5 wt %, respectively (Figure 4a).

Comparing the experimental formulation results (i.e., LC for A-pPentOx-A > A-pBuOx-A > A-pPrOx) with the theoretical polymer–drug compatibility results (i.e., pPrOx > pBuOx > pPentOx) obtained by solubility parameters, it is clear that the HSPs gave unsatisfactory results, in fact yielded predictions completely opposite to the obtained results. The predictive power of solubility parameters (δ_d_, δ_p_, δ_h_, and δ_total_) is based on relatively straightforward mathematical calculations,^53,65^ limiting the precise prediction of the overall polarity and hydrogen-bonding ability of molecules. In addition, the interplay between hydrophilic/lipophilic domains and cargo in a solution is much more complex, which can lead to deviations from predicted interactions^17−20,67^ compromising the predictive power of solubility parameters.^41^ We are aware that in the past, several authors have claimed that HSPs are well suited for the prediction of polymer–drug compatibilities,^62,68^ including in POx-based block copoylmers,^69^ but our results presented here and before^34,41^ clearly show that one has to be very careful not to overgeneralize. While it is obvious that HSPs obtained by group contribution methods are not suitable for compatibility prediction in the present systems, it would be interesting to investigate other approaches such as molecular dynamics or active learning.^63^

### Stability Studies

For pharmaceutical development and commercialization, the stability of the formulation is one of the basic requirements. To investigate the shelf life of BT44 formulations, the freshly prepared formulations were stored at room temperature with the initial precipitate (if any), and samples were collected at day 0, 1, 5, and 15. Previously, we had observed that the A-pBuOx-A/PTX formulation exhibited an excellent stability for several months;^32,58^ however, in a few cases the formulation with similar amphiphiles was found to be much less stable.^36,60^ The A-pPrOx-A/BT44 formulation was not included in the stability study because of the poor drug-loading capacity (i.e., LC ≈ 0.5 wt %). Unlike the stable A-pBuOx-A/PTX formulation, the BT44 formulation with A-pBuOx-A was found to be less stable, and in the course of time a gradual decrease in LC was observed in all the tested ratios, e.g., the highest LC obtained with the A-pBuOx-A/BT44 feed of 10/8 g/L decreased from 19 % (at day 0) to 7 wt % (at day 15) (Figure 4c, red bars).

Interestingly, in the case of A-pPentOx-A, a direct relationship between LC and formulation stability was observed, i.e., the formulations with high LC were found to be relatively more stable (Figure 4d). The formulations with 2 g/L of BT44 feed precipitated completely within 24 h, while the formulations with 4–6 and 8–10 g/L of BT44 feed precipitated completely by day 5 and 15, respectively. The present findings are opposite to our previous results, where the mitotane (MT)-based formulation with A-pBuOx-A polymer (for treatment of adrenocortical carcinoma) was found to be more stable at lower MT feed (2 and 4 g/L). However, at a certain threshold MT feed (≥6 g/L), a rapid crystallization of MT was observed corroborated by a clear melting peak in the DSC thermogram of the precipitate.^34^ To investigate the reasons for BT44/A-pPentOx-A/formulation instability, i.e., whether BT44 crystallizes or the formulation is colloidally unstable, the formulations were further characterized by the following techniques.

### Physicochemical Characterization of the BT44/POx Formulation

As previously explained, the interactions between drugs and the hydrophilic corona of polymer micelles are becoming more evident, which can lead to either high drug loading^20,67^ or colloidal instability of the formulations^51^ but may also affect endocytosis.^22,70^ The limited colloidal stability is evidenced by the fact that the resultant precipitate contains not only the drug but also the otherwise highly water-soluble polymer.^18,33^ To evaluate this for the present formulations, the A-pPentOx-A/BT44 (100/20 g/L) formulation was prepared. After centrifugation, the supernatant and the sediment were collected separately and lyophilized. For better comparison, the ^1^H NMR spectra of the pristine BT44, the plain A-pPentOx-A triblock polymer, and the (lyophilized) precipitate in chloroform-D (CDCl_3_) as nonselective solvent (no micelles formation) were obtained (Figure S4). The ^1^H NMR spectra of the plain polymer clearly presented all the signals, suggesting that the polymer existed as unimers in nonaggregated form (Figure S4, green spectra), and it was also evident that the precipitate contained not only the drug but also the hydrophilic polymer (Figure S4, red spectra), corroborating our previous findings.^19,33,57^ However, the precipitate was significantly enriched in BT44 compared to the polymer (4/1 drug/polymer ratio).

To gain more insights into the BT44 formulations, ^1^H NMR experiments were further performed using deuterium oxide (D_2_O) as a selective solvent (micelles formation). Initially, the ^1^H NMR spectra of all three plain triblock copolymers used in this study were obtained (at a concentration of 10 g/L) (Figure S5). The A-pPrOx-A copolymer exhibited all the signals from the hydrophilic as well as the hydrophobic block (Figure S5, blue spectra, signals 1–6). This shows that the polymer existed as unimers in D_2_O because of very low amphiphilic contrast, unable to self-assemble into micelles. This could be expected, as the pyrene assay previously also indicted no formation of micelles at 10 g/L concentration for A-pPrOx-A triblock copolymer.^41^ Lübtow et al. also observed a similar behavior for poly(2-*n*-propyl-2-oxazine)-based triblock copolymer (A-pPrOzi-A), and the micellization was only induced in the presence of a hydrophobic molecule, i.e., CUR. Additionally, at ultrahigh CUR loading (LC > 50 wt %), the signals from the hydrophobic block were still visible, albeit with lower intensities (in D_2_O), while the CUR signals were completely attenuated.^33^ The ^1^H NMR spectroscopy also revealed a similar pattern for PTX^67^ and atorvastatin (ATV)^57^ loaded A-pBuOx-A and A-pBuOzi-A ((poly(2-*n*-butyl-2-oxazine)) micellar formulations, respectively. However, in the absence of the drug, small-angle neutron scattering (SANS) revealed that the plain A-pBuOzi-A polymer existed as spherical self-assemblies without a well-defined core–shell architecture. In contrast, the plain A-pBuOx-A displayed a core–shell morphology.^20^ The ^1^H NMR spectra of A-pBuOx-A^41^ also exhibited all the respective signals including the hydrophobic block (Figure S5, green spectra, signals 1–7). Herein, the ^1^H NMR spectra of the plain A-pPentOx-A (Figure S6, green spectra) and the lyophilized BT44 formulations (Figure S6, red spectra) were also obtained in D_2_O. In both spectra, the signals corresponding to the hydrophobic block were completely absent (Figure S5, red spectra, signals 3, 5, 6, and 7), and no signals from the BT44 were visible in the formulation. In contrast, Hiller et al. observed all the representative signals (^1^H NMR) from the plain diblock copolymer, i.e., pMeOx_17_-pPentOx_3_, including the signals from hydrophobic pPentOx in D_2_O.^71^ However, in comparison to the present A-pPentOx-A triblock copolymer, Hiller et al. investigated a very short hydrophobic block (three repeat units), resulting in very low amphiphilic contrast. The complete disappearance of signals from the hydrophobic block in plain A-pPentOx-A triblock, presented herein, indicates a short transverse relaxation time (*T*_2_) and suggests micelles with a rigid (solidlike) core both in the presence and in the absence of BT44. The direct comparison of A-pBuOx-A and A-pPentOx-A micellar formulations utilizing analytical tools like solid-state NMR^18,67^ and SANS could give us a much deeper understanding of polymer drug interactions resulting in the different micellar structures and dynamics; however, such analysis is beyond the scope of the current contribution. In order to investigate the reasons for the poor stability of the A-pPentOx-A/BT44 formulation, DSC analysis was performed to evaluate whether the precipitation is associated with BT44 crystallization similar to the previously reported MT-formulation^34^ or because of colloidal instability and the resulting agglomeration and sedimentation of the micelles. Initially, the DSC thermogram of the pristine BT44 was obtained to determine the melting point (Figure S7a). The sharp endothermic peak at 146 °C in the first heating cycle indicated the crystalline nature of BT44 (Figure S7a, black curve), while the DSC traces in the second and third heating cycles did not show any melting peak, as BT44 remained in its amorphous form with a *T*_g_ of 75 °C (Figure S7a, green vertical line). In this regard, only the first heating cycle in the DSC thermograms of pure BT44 and the resultant precipitate in the formulation (i.e., A-pPentOx-A/BT44 at 10/4 g/L feed) at day 5 were compared (after lyophilization). Unlike for pristine BT44, no melting peak was observed in the precipitate at day 5, suggesting an amorphous nature of BT44 (Figure S7b, red curve), corroborating our previous findings^32,42^ that the poor stability can be correlated to micellar agglomeration and sedimentation.

**Figure 5:**
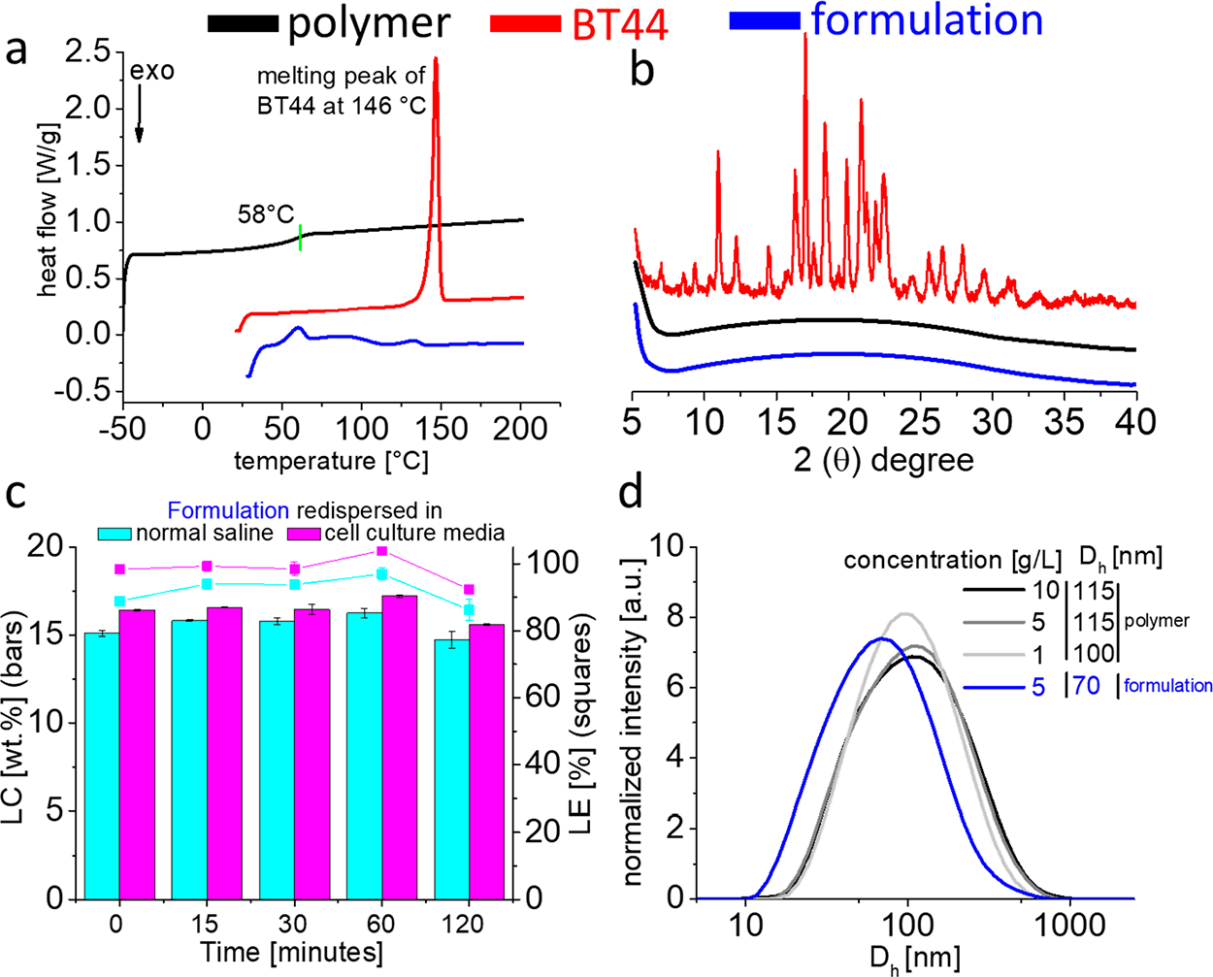
(a) DSC thermograms (at a heating rate of 10 K/min) of pristine BT44 (red, first heating cycle, sharp melting peak at 146 °C), plain polymer (black, second heating cycle, *T*_g_ at 58 °C), and lyophilized formulation (blue, first heating cycle, absence of a melting peak for BT44), respectively, after 15 days of storage at room temperature. (b) Powder XRD spectra of pristine BT44 (red), plain polymer (black), and lyophilized formulation (blue) after 1 month of storage at room temperature. (c) Redispersion of lyophilized formulation (polymer/BT44 100/20 g/L) after 30 days of storage at room temperature in normal saline (blue) and cell culture media (magenta) for short-term stability studies from time 0 to 120 min. Data are given as means ± SD (*n* = 3). (d) The size distribution by intensity (measured at 173° scattering vector) of plain polymer (10, 5, and 1 g/L) and BT44 formulation (5 g/L) redispersed in normal saline (filtered through a 0.45 μm PVDF filter).

In pharmaceutical product design, the formulation instability is a serious risk factor which can limit their widespread use. Lyophilization is generally a primary strategy to improve the shelf life of a drug product. Therefore, the A-pPentOx-A/BT44 100/20 g/L formulation was prepared (Figure 4b) and lyophilized. During storage, the physicochemical stability of the lyophilized formulation is also one of the quality attributes that should be evaluated. Such formulations have the tendency to crystallize during handling, storage, or transportation. In order to investigate this, the DSC thermograms of the plain polymer, pristine BT44, and the lyophilized formulation after 15 days of storage at room temperature were compared (Figure 5a). The absence of a melting peak (at 146 °C) in the first heating cycle indicated again that the BT44 remained as an amorphous form in the lyophilized formulation (Figure 5a blue curve). In the second and third heating cycles, unlike for pristine BT44 (*T*_g_ at 75 °C, Figure S7a) a single *T*_g_ was observed at 58 °C (Figure S8) which corresponds to the *T*_g_ of plain A-pPentOx-A polymer, indicative of the complete miscibility of the drug and polymer.

For further analysis, the pristine BT44, plain polymer, and lyophilized formulation were analyzed by powder XRD after 30 days of storage at room temperature (Figure 5b). The diffractograms of the pristine BT44 showed multiple peaks in the 2Θ range of 10–35° showing the crystalline nature of BT44 (Figure 5b, red curve). In contrast, the broad halos without any traces of peaks in the plain polymer and lyophilized formulation indicate their amorphous nature (Figure 5b , black and blue curve, respectively). This preliminary stability study using DSC and powder XRD analysis suggests that such formulations can be stored reasonably well, as a lyophilized powder without a high risk of recrystallization. However, more detailed studies at different temperatures and relative humidities will be needed to complete the picture. In addition, the analysis of traces of crystallinity via multimodal nonlinear imaging would also be of interest.^72^

For lyophilized formulations, the complete redispersion or aqueous reconstitution is also a very important step for parenteral administration. In addition, the evaluation of the short-term stability of the redispersed formulation is relevant in practical settings. The final redispersed formulation should remain stable for the desired period of time to conveniently allow for parenteral administration (injection or infusion). Previously, lyophilized POx-based PTX, CUR, ATV, and MT formulations (without any additional cryo-/lyoprotectants) displayed excellent redispersibility before use.^33,34,57^ In this regard, A-pPentOx-A/BT44 100/20 g/L lyophilized formulations (after storage at room temperature for 30 days) were redispersed in normal saline and cell culture media (without serum) and quantified at time 0, 15, 30, 60, and 120 min by HPLC to determine the short-term storage (Figure 5c). Upon redispersion (at *t* = 0 min) with normal saline, initially a minor decrease in LC (i.e., 16 to 15 wt %) was observed, while with cell culture media, 100% redispersibility was noticed (LC ≈ 16 wt %). In the time course of 120 min, the LC of normal saline and cell culture media-based formulations decreased slightly to 14 and 15 wt %, respectively, and accordingly, a minor precipitation was noted. From the redispersion results, it can be concluded that A-pPentOx-A/BT44 formulations can be stored as lyophilized powder but need to be used immediately after redispersion.

To estimate the size of self-assemblies, redispersed formulations (in normal saline) were characterized by dynamic light scattering (at 173°) at room temperature (Figure 5d). Initially, the self-assemblies of plain A-pPentOx-A polymer at different concentrations (10, 5, and 1 g/L) were measured, and the size (hydrodynamic diameter, *D*_h_) fell into the range of 100–115 nm. However, upon the incorporation of BT44 (5 g/L), the *D*_h_ decreased to approximately 70 nm indicative of BT44-induced reorganization and compaction of the self-assemblies (Figure 5d, blue curve). However, it should be clear that the sizes of these self-assemblies are beyond the size to be expected for simple, spherical polymer micelles, as the theoretical extended chain length of the polymers is well below 40 nm.

**Figure 6:**
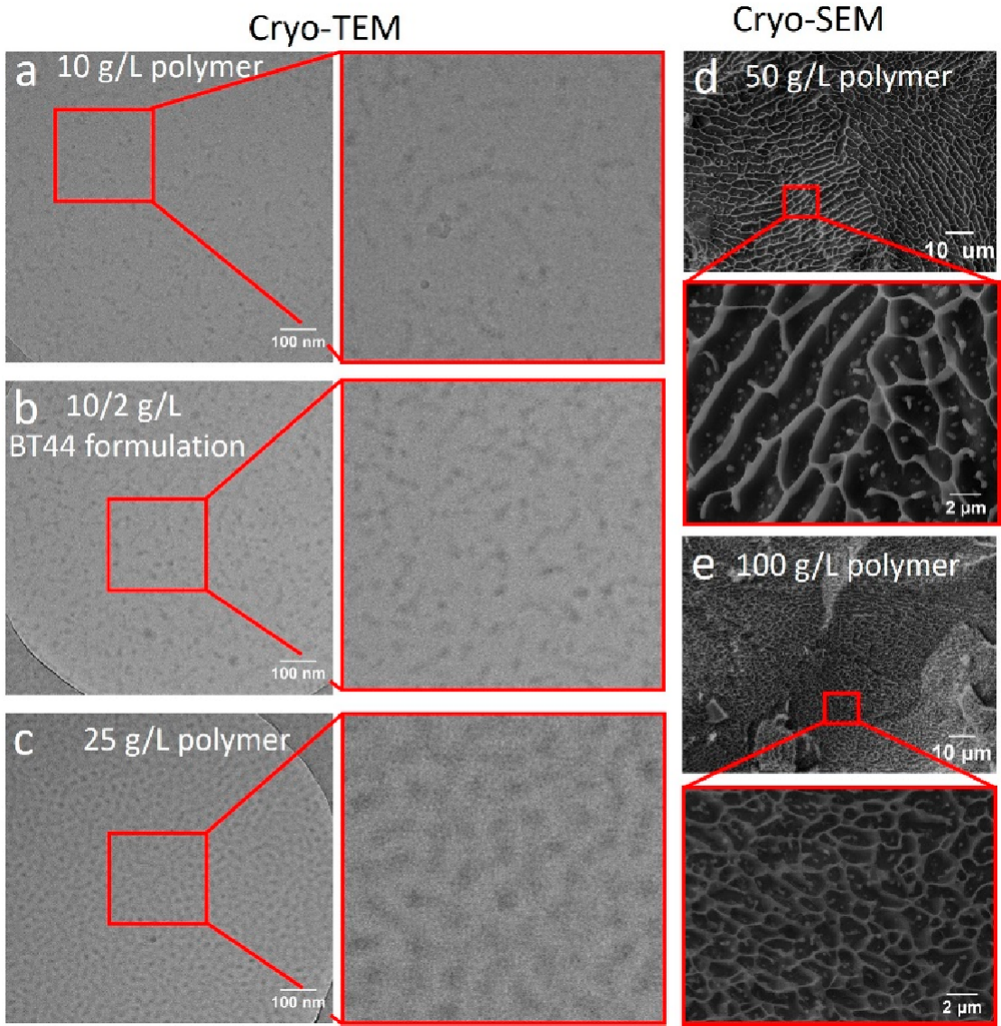
Cryo-transmission electron microscopy analysis (cryo-TEM) of (a) 10 g/L plain A-pPentOx-A polymer, (b) 10/2 g/L A-pPentOx-A/BT44 formulation, (c) 25 g/L plain A-pPentOx-A polymer with a respectively closer view for better visualization of wormlike structures. The cryo-scanning electron microscopy (cryo-SEM) analysis of (d) 50 and (e) 100 g/L plain A-pPentOx-A polymer (acceleration voltage ETH: 8 kV).

To gain more insights into the size and morphology of the assemblies, initially, we visualized the plain polymer solution (at concentration of 10 and 25 g/L) and polymer/BT44 (10/2 g/L) formulation by cryo-TEM. The results revealed the presence of a few short wormlike assemblies (Figure 6a). This can explain the higher *D*_h_ observed by DLS, i.e., 100–115 nm (at 10 g/L plain polymer solution). No notable differences were observed in the morphology, when the plain polymer was compared to the BT44 formulation (at polymer/BT44 10/2 g/L) (Figure 6b). When the polymer concentration increased from 10 to 25 g/L, the overall population of the worms also increased, and the whole grid seemed to be saturated with these assemblies (Figure 6c). The alignment, range of intermediate structures, and further branching between the worms are indicative of a dense network formation. Generally, the transition to higher-order assemblies often correlates with an increase in viscosity. For example, by cryo-TEM, Hahn et al. observed the morphological transition from spherical micelles to a wormlike network causing the macroscopic gelation for poly(2-phenyl-2-oxazine)-based A-B-A (A-pPheOzi_15_-A) triblock copolymer (at 200 g/L concentration).^73^ Here, both the A-pPentOx-A/BT44 formulation and the plain polymer solution appeared as free-flowing liquids at all the tested concentrations. To obtain further insights into the morphology of A-pPentOx-A plain polymer solutions at 50 and 100 g/L (at which TEM is not feasible), the visualization was performed by cryo-SEM. Similar to the previously reported case of A-pPheOzi_5_-A hydrogel,^73^ the 50 g/L A-pPentOx-A polymer solution (without forming hydrogel) also exhibited lamellae-like structures (Figure 6d), which were further condensed by increasing the polymer concentration to 100 g/L (Figure 6e). This further condensation led to structural transition to a honeycomb-like structure. To the best of our knowledge, this is the first study presenting the wormlike morphological details of any pPentOx-based triblock copolymer and its drug formulations. However, previously the wormlike structures have been reported for A-pPrOzi-A/CUR^33^ and A-pBuOx-A/cisplatin/etoposide formulations,^46^ while in the presence of PTX, A-pBuOx-A exhibited spherical/raspberry-like micelles.^24^ The plain A-pPrOzi-A existed as unimers,^33^ while plain A-pBuOx-A polymer solution exhibited spherical micelles along with the presence of a few wormlike assemblies.^24^ Recently, depending upon the drug feed, Lim et al. observed the transition of freshly prepared A-pBuOx-A/Olaparib spherical micelles (*D*_h_ ≈ 10–30 nm) to wormlike structures (*D*_h_ ≈ 200 nm) in the time span of 72 h.^74^ The authors further suggested that the spherical micelles can be therapeutically more efficient for the systemic delivery of anticancer drugs when compared to wormlike assemblies. However, this should be verified on a case-by-case basis because the nature of the hydrophobic block, the overall hydrophilic lipophilic balance, the chemical nature of the drug, and the drug feed are the major driving forces for such kinds of transitions, which might result in loosely or densely packed micellar architectures, resulting in rapid or slow release of drugs.

### *In Vitro* and *In Vivo* Studies

#### Assessment of Polymer Cytocompatibility by Alamar Blue Assay

**Figure 7:**
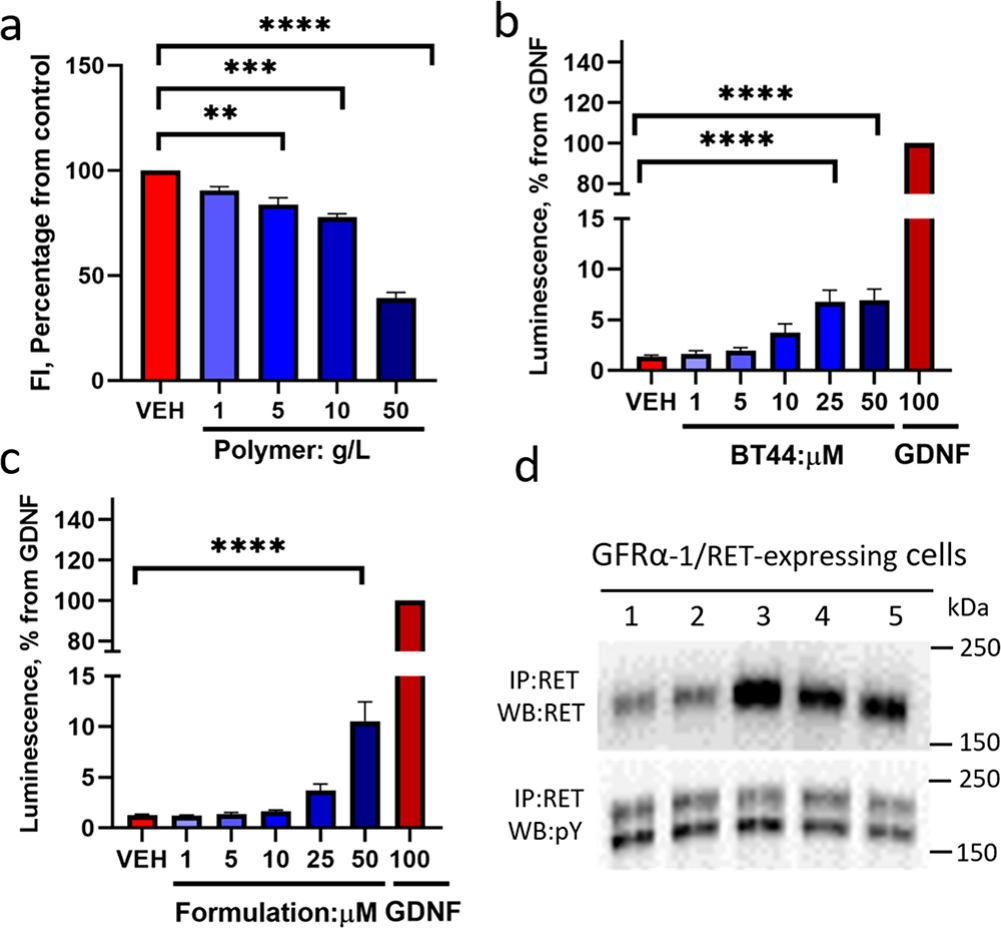
(a) Cytotoxicity study of the plain polymer (A-pPentOx-A) at various concentrations, studied by Alamar blue assay after 72 h of incubation with MG87 RET cells. The quantitative data are presented as mean ± SEM (VEH; vehicle, cell culture media, FI; fluorescence intensity, ANOVA with Dunnett’s *post hoc* test compared to vehicle, *n* = 4; ** *p* < 0.01, *** *p* < 0.001, **** *p* < 0.0001). (b) BT44 (in 1% DMSO) and (c) nanoformulated BT44 (at various concentrations) induced luciferase activity in reporter gene-based assay. (d) Assessment of RET phosphorylation by Western blotting in GFRα1-transfected MG87 RET cells. Lanes 1, 2, 3, 4, and 5 are VEH (1% DMSO in DMEM), plain polymer (0.64 mg/mL, similar to 79 μM), BT44 nanoformulation (100 μM), pristine BT44 dissolved in 1% DMSO (100 μM), and GDNF (6.6 nM), respectively. The quantitative data are presented as mean ± SEM. Concentrations provided are in ng/mL for GDNF and μM for polymer, nanoformulation, and BT44. (IP; immunoprecipitation, WB; Western blotting. **** *p* < 0.0001, one-way ANOVA with Dunnett’s *post hoc* test, number of experiment (*n* = 2–4).)

To be suitable for preclinical development and clinical translation, the excipients used for formulation development must be nontoxic. We, therefore, evaluated the cytocompatibility of the polymer (A-pPentOx-A) *in vitro* using Alamar blue assay. However, the POx-based amphiphiles have been repeatedly established to be cytocompatible *in vitro* and *in vivo*.^24,33−35^ In this regard, we have also performed cytocompatibility studies of the A-pPentOx-A-based triblock copolymer in MG87 RET cells. The cells were exposed to 1–50 g/L of polymer for 72 h. We observed a polymer concentration dependent decrease in cell viability with respect to untreated cells (Figure 7a). At 1 g/L of polymer, no significant reduction in cell viability was observed, while a minor reduction was observed in the cells treated with 10 g/L of polymer (*p* = 0.0001, ANOVA with Dunnett’s *post hoc*). In contrast, at 50 g/L of polymer, the cell viability dropped well below 50% of the control (*p* < 0.00001, ANOVA with Dunnett’s *post hoc*). Previously, we have seen no considerable cytotoxicity for a variety of POx-based AB and ABA triblock copolymers even at such high concentration.^33,34,57^ However, it should be kept in mind that this is expected to be dependent on the cell type, and the used concentrations are relatively high, exceeding commonly investigated concentration ranges by 1–2 orders of magnitude. In addition, some discrepancies in the cytotoxicity assessment might also be related to the differences in experimental methods (MTT/WST vs Alamar Blue assay).

#### Biological Activity of the BT44 Formulation in Immortalized Cells

The GDNF family ligands signal through the GFRα/RET complex.^75^ Previously, it has been shown that RET agonists BT13 and BT44 stimulate RET phosphorylation and RET-dependent downstream intracellular signaling.^4,5,7,75^ Therefore, in the present study we evaluated the ability of nanoformulated BT44 to activate RET and RET-dependent downstream signaling in MG87 RET fibroblasts transfected with GFRα1 by Western blot and using a reporter gene-based assay.

The BT44 nanoformulation increased the luciferase activity by 8.4-fold (*p* < 0.0001, ANOVA with Dunnett’s *post hoc*) at 50 μM concentration (Figure 7c) in the reporter cell line. In line with previously published results, nonformulated BT44 (Figure 7b) increased the luciferase activity at both 25 and 50 μM concentration by 8.9-fold (*p* < 0.0001, ANOVA with Dunnett’s *post hoc*). The GDNF was used as a positive control, and data were normalized to the signal elicited by GDNF. In line with reporter gene-based assay results, nanoformulated BT44 increased the level of phosphorylated RET in GFRα1-transfected MG87 RET cells compared to both vehicle and plain polymer (Figure 7d). We also quantified the band intensities of both phosphorylated RET and pan RET from the Western blot imaged obtained in two independent experiments (Figure S9). Repeated-measurements ANOVA analysis revealed the difference between the treatment groups (*p* = 0.0439), and on the basis of *post hoc* analysis, BT44 nanoformulation increased the level of RET phosphorylation by 3.6-fold (*p* = 0.0469, ANOVA with Dunnett’s *post hoc* test). These data indicate that the POx-based BT44 nanoformulation retained the ability to activate RET and downstream signaling pathways.

#### Neuroprotective Properties of the BT44 Formulation in Dopamine Neurons

**Figure 8:**
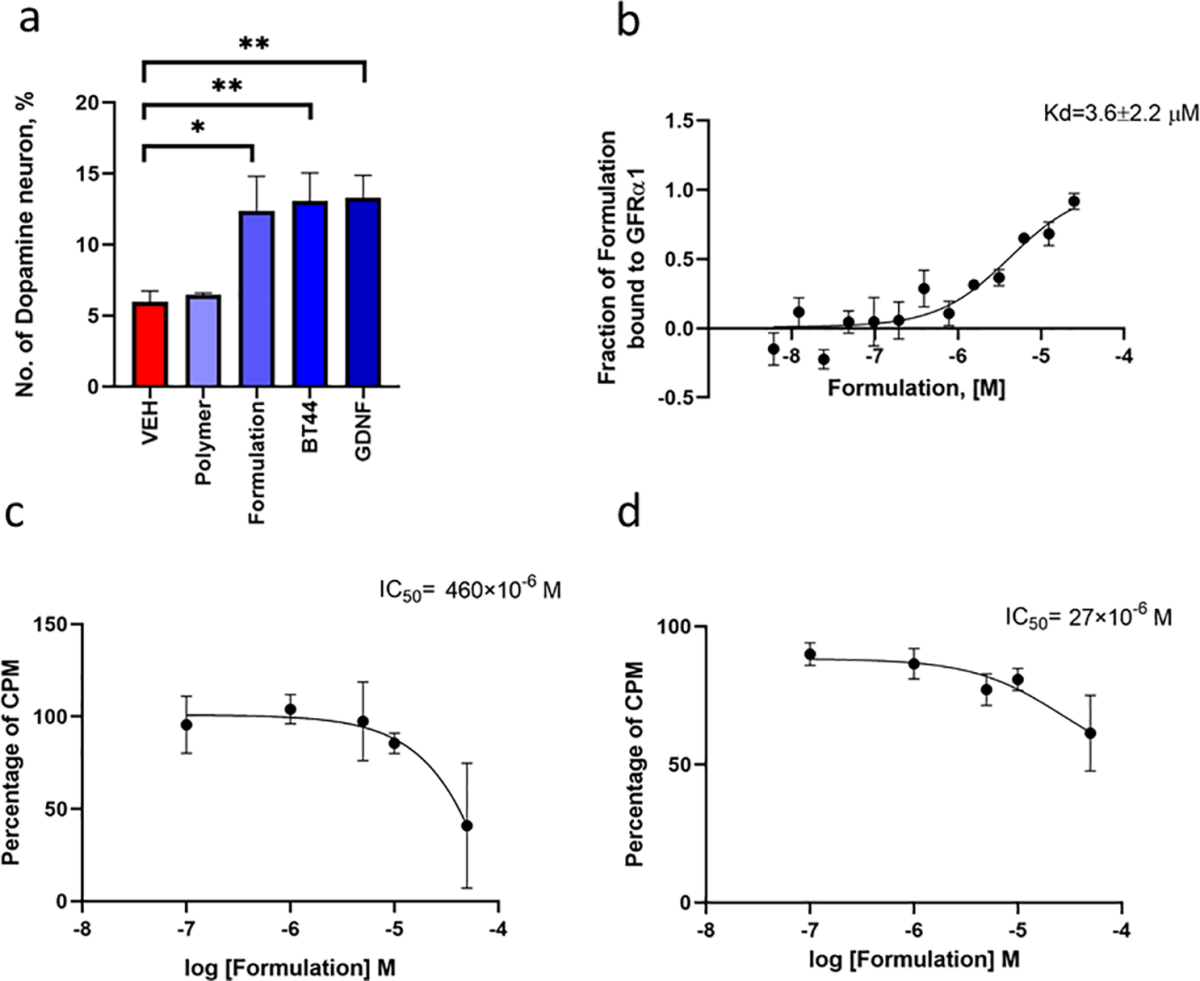
(a) BT44 nanoformulation protected cultured dopamine neurons from MMP+ neurotoxin-induced cell death. The number of TH-positive cells in the wild-type midbrain cultures exposed with MPP+, after 6 days *in vitro* culture, were exposed to vehicle, plain polymer (0.0064 mg/mL, similar to 790 nM), nanoformulated BT44 (100 nM), pristine BT44 (100 nM in 1% DMSO), or GDNF (3.3 nM), respectively, for 48 h. The results are normalized to the total number of cells in the culture and presented as a percentage of vehicle (1% DMSO in DMEM F12 media) treated samples (average from 4 independent experiments). The quantitative data are presented as mean ± SEM (VEH; Vehicle, 1% DMSO in DMEM F12 media. * *p* < 0.05, ** *p* < 0.01, one-way ANOVA with Dunnett’s *post hoc* test). (b) The binding of nanoformulated BT44 (0–10 μM) to GFRα1 (20 nM) monitored by MST. (c) and (d) Displacement of 50 pM ^125^I-GDNF from GFR α1 (c), GFR α1/RET complex, and (d) by nanoformulated BT44. Each data points represents mean ± SEM of 2–4 independent repeats. The binding isotherms were fitted using nonlinear regression.

Both BT13 and BT44 were previously shown to promote the survival of cultured dopamine neurons and also protect these neurons from dopamine neurotoxin-induced cell death.^5,8^ Therefore, we also tested the ability of nanoformulated BT44 to protect dopamine neurons against *N*-methyl-4-phenylpyridine (MMP+)-induced cell death. MMP+ is a neurotoxin that inhibits the complex I of the mitochondria leading to the depletion of adenosine triphosphate (ATP) and eventually causing cell death. At 100 nM, nanoformulated BT44 increased the number of TH-positive neurons by 1.9-fold (*p* = 0.0103), which is comparable to the effect produced by BT44 dissolved in 1% DMSO (2.2-fold increase, *p* = 0.0050) and GDNF (2.2-fold increase, *p* = 0.0040) (*p* = 0.0013, ANOVA with Dunnett’s *post hoc* test for all comparisons) (Figure 8a). Thus, the pPentOx-based BT44 nanoformulation did not negatively affect the neuroprotective properties of BT44.

#### Interaction of the BT44 Formulation with GDNF Receptors

Further, we studied the binding of nanoformulated BT44 with GFRα1 and GFRα1/RET using MST and cell-based iodinated GDNF displacement assay. The binding of neurotrophic factors GDNF to GFRα1 and the activation of RET receptors have been investigated in various laboratories.^76^ The GDNF binds to the soluble form of GFRα1 with *K*_d_ of 630 pM as shown by surface plasmon resonance binding studies.^77^ Using MST assay, we observed a somewhat lower binding affinity of GDNF to GFRα1 (*K*_d_ = 260 nM), thus validating the assay. The POx-based BT44 formulation also bound to GFRα1, although with much lower affinity (*K*_d_ = 3.6 ± 2.2 μM) (Figure 8b). The GDNF is a rather big protein molecule which forms multiple contacts with GFRα1, while compounds belonging to the BT family make only a few hydrogen bonds and other contacts with amino acid residues of GFRa1.^78^ Therefore, the higher *K*_d_ of BT44 compared to that of GDNF in GFRa1 binding assay is to be expected. Importantly, binding of the BT44 formulation to GFRα1 in the presence of RET was not observed, and rather aberrant MST traces were seen owing to protein aggregation. Here, we speculate that aggregates observed in this assay might be due to the complex formation between GFRα1, the nanoformulation, and RET. Further studies are needed to verify this hypothesis.

We also performed competition-binding studies with formulated BT44 in cells expressing GFRα1 or GFRα1/RET. In this assay, nanoformulated BT44 displaced radiolabeled GDNF with an IC_50_ value of 460 × 10^–6^ M in GFRα1-transfected cells (Figure 8c) and 27 × 10^–6^ M (Figure 8d) in GFRα1/RET-transfected cells. When GFRα1 was coexpressed with RET at a DNA ratio of 1:1, the potency of the nanoformulated BT44 in ^125^I-GDNF assay was higher compared to that observed in the cells expressing only GFRα1. A similar trend was also observed for GDNF in GFRα1-expressing cells, wherein unlabeled GDNF displaced ^125^I-GDNF with an IC_50_ value of 1.9 ± 0.2 × 10^–9^ M, while in GFRα1/RET-transfected cells (1:1 DNA ratio) an IC_50_ of 10.6 ± 2.1 × 10^–12^ M for the high-affinity site and 2.3 ± 1.1 × 10^–9^ M for the low-affinity site^77^ was found. Overall, our *in vitro* studies suggest that nanoformulated BT44 has similar effects to that of free BT44 and GDNF.

#### *In Vivo* Evaluation of Absorption, BBB Penetration, and Tolerability of the BT44 Formulation

In previous *in vivo* studies, propylene glycol (PG) was used as a vehicle for the BT44 administration, owing to the poor aqueous solubility of BT44.^7,8^ In rats, PG did not show any significant toxicity after both subcutaneous and intracranial administration. However, the absorption of BT44 into the bloodstream after subcutaneous injection of the BT44 dissolved in PG was found to be rather low.^7^ After intravenous administration, a portion of BT44 (approximately 18%) also penetrated through the BBB.^8^ Herein, we investigated whether a higher absorption and potentially even better BBB penetration can be achieved with the nanoformulated BT44 after subcutaneous administration.

**Figure 9:**
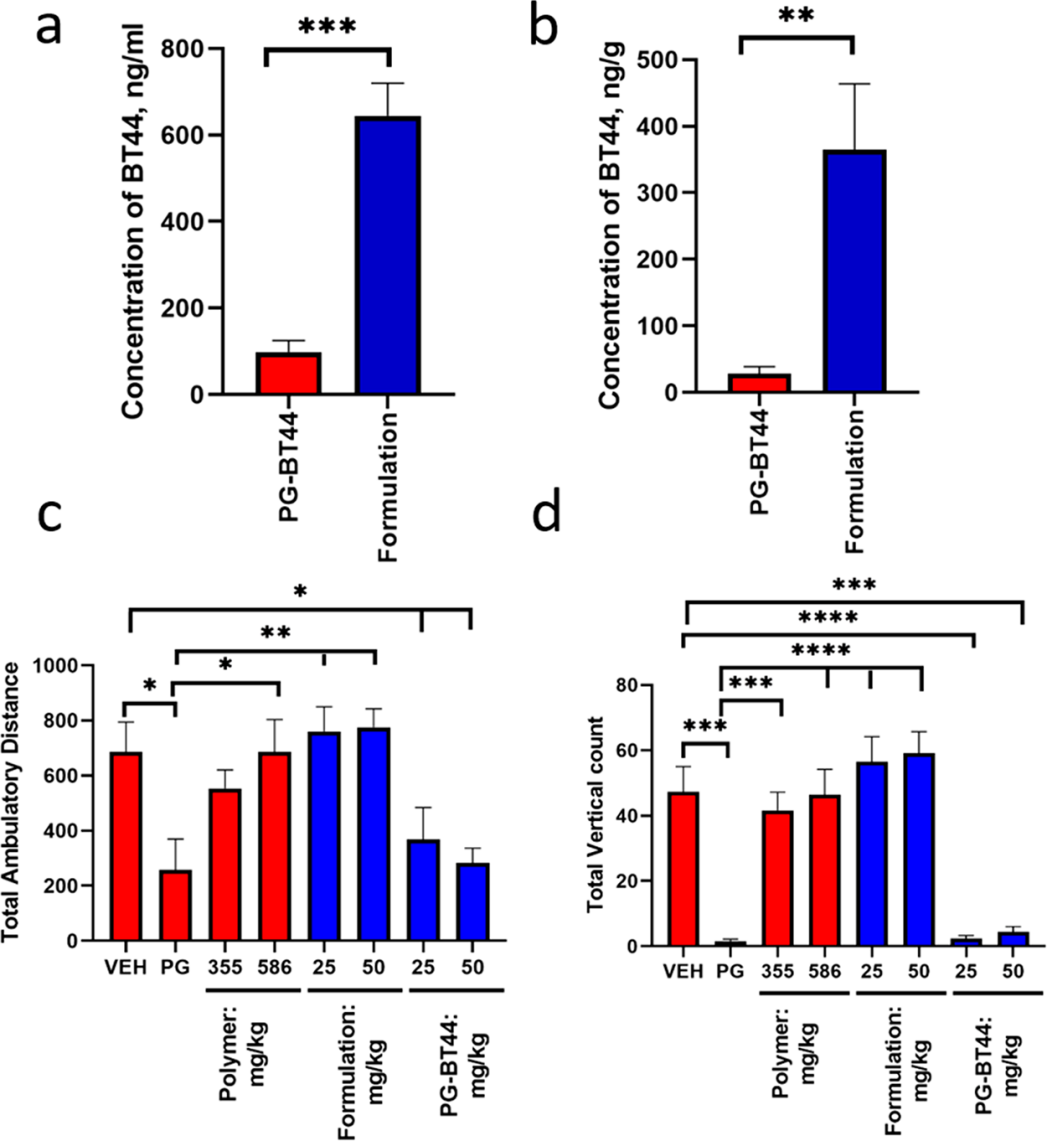
Plasma and brain concentrations of the BT44 formulation and tolerability studies in mice after subcutaneous administration. The plasma (a) and brain (b) concentration of BT44 after 1 h propylene glycol (PG)-solubilized BT44 and nanoformulation injection. The tolerability of the formulation was monitored using behavioral parameters such as the total ambulatory distance traveled (c) and the total vertical count (d) in the open field behavioral test. Each data point represents mean ± SEM of 4–5 mice per group. * *p* < 0.05, ** *p* < 0.01, *** *p* < 0.001, **** *p* < 0.0001, one-way ANOVA with Dunnett’s *post hoc* test.

The plasma and brain concentrations of BT44 were measured 1 h postinjection and found to be significantly higher in the case of nanoformulated BT44 compared to PG-based solution, i.e., 6.6-fold, *p* = 0.0001 and 13-fold, *p* = 0.0094, respectively (Figure 9a and 9b). Apparently, nanoformulated BT44 not only increased the absorption of BT44 into bloodstream but also enhanced the BBB penetration of the compound in mice receiving nanoformulated BT44, both plasma and brain concentrations of the compound were higher compared to mice treated with pristine BT44, but the plasma concentration was 6.5-fold higher, while the brain concentration was 12.8-fold higher. Thus, the relative increase of BT44 concentration using the nanoformulation was approximately two times higher in brain than in plasma. Very recently, Hwang et al. also observed a similar effect by using an A-pBuOx-A/vismodegib (VSM)-based nanoformulation, as the VSM concentration was found to be much higher in the medulloblastoma and forebrain of the mice compared to the free drug. The authors hypothesized that the encapsulation of VSM in nanocarriers reduced its interaction with plasma proteins, hence increasing the availability of free VSM and leading to relatively higher transport across BBB.^26^ Another possibility could be interference with drug efflux pumps which are prevalent in the BBB, and their role in restricting the brain entry of multiple drugs is widely accepted.^79^ However, further studies are needed to support or reject this hypothesis. Interestingly, using a chemically totally unrelated amphiphilic copolymer, Zentel and co-workers reported higher transport across the BBB for nanoformulated domperidone and rhodamine 123, which normally have poor BBB permeability.^80−82^ In any case, the observed increase in BBB penetration is highly beneficial for CNS-targeted drugs.

Finally, the tolerability of nanoformulated BT44 in mice was also evaluated. In this regard, the locomotor and exploratory activity were assessed by the open field behavioral test. Additionally, the general condition of the animals was visually monitored. To our surprise, and in contrast to previous experimental observations in rats, the vehicle, i.e., PG, negatively impacted the behavior of the experimental animals, i.e., mice. The statistically significant differences were observed in motor function (both total ambulatory distance traveled and total vertical counts) between the treatment groups (*p* = < 0.0001, one-way ANOVA) 1 h postadministration of tested substances. The total ambulatory distance traveled by mice treated with PG (*p* = 0.0393) alone and PG-BT44 both at 25 (*p* = 0.0422) and at 50 mg/kg (*p* = 0.0381) was significantly reduced compared to this characteristic for mice treated with saline (Figure 9c). Further, vertical counts were also affected by PG alone (*p* = 0.0001) or in combination with 25 mg/kg (*p* < 0.0001) or 50 mg/kg (*p* = 0.0001) of BT44 (Figure 9d). The reduction in motor activity was also visually very clear in home cages. The mice receiving PG (either alone or in combination with BT44) remained immobile and practically nonreactive to handling. The effect was temporary, and within a few hours postinjection (2–6 h), the mobility of animals was gradually restored. All animals that remained immobile for ≥2 h received intraperitoneal injection of 1 mL of saline to prevent dehydration. All animals were visually normal at 24 h post-treatment. Notably, the reported no-observed-effect level (NOEL) and maximum tolerated dose (MTD) for PG in mice when injected intravenously were 1.036 and 1.554 g/kg, respectively.^83^ Toxicity manifestations, such as hind limb ataxia, hind limb muscle contraction, and hemolysis, were observed when the dose of PG exceeded MTD. According to the information from the PubChem database, the lethal dose 50 (LD_50_) of PG in mice and rats (after subcutaneous injection) is equal to 17.4 and 28.8 g/kg, respectively. In the current study, the dose of PG administered subcutaneously constituted 10.3 g/kg. This is much below LD_50_ but seems to be sufficient to produce notable adverse effects in mice, even though in a previous study, no such effects were observed in rats.^6^ Thus, there are interspecies differences in sensitivity to PG, which is in line with published data (PubChem). Noteworthy, there are several reports indicating PG toxicity in humans.^84^ Further, PG was found to cause neuroapoptosis in the developing mouse central nervous system.^85^

In comparison, the nanoformulated BT44 (at 25 and 50 mg/kg) and plain polymer (at corresponding doses of 355 and 586 mg/kg) produced no significant changes in evaluated behavioral parameters (total ambulatory distance and vertical counts) after subcutaneous injection (Figure 9c and d). The animals receiving plain polymer or BT44-loaded POx formulation freely moved in home cages and were responsive to the investigator’s manipulations. These data suggest that the plain polymer excipient and nanoformulated BT44 are safe *in vivo* and could be used for future preclinical development.

## Conclusion

Herein, we present the first micellar nanoformulation of BT44 with an ultrahigh drug loading capacity. The extent and pattern of compatibility between polymer and drug when considering Hansen solubility parameters between BT44 and three amphiphiles were not in accordance with the formulation results. As has been described before for other drugs, Hansen solubility parameters are not the appropriate choice for estimating compatibility in a POx-based system, especially considering the fact that depending upon the nature and overall load of guest molecules, the hydrophilic pMeOx plays an important role in the solubility as well as the stability of the entire system.^18,20,67^ The POx-based triblock copolymer, i.e., A-pPentOx-A, gave an ultrahigh drug loading of around 47 wt %. The developed formulations underwent detailed physicochemical and biological characterization. The formulation can be stored as lyophilized powder ready for redispersion. The formulated BT44 bound to its cellular receptor and retained its biological activity in immortalized cells and its neuroprotective properties in dopamine neurons. Furthermore, the absorption of formulated BT44 into the bloodstream and its blood–brain barrier penetration properties were significantly improved compared to the conventional preparation of BT44 in PG. We did not observe acute toxicity of either the POx polymer or the BT44/POx formulation in mice. Similar to previously reported POx-based triblock copolymer formulations,^26,35,45,46^ we anticipate that the A-pPentOx-A/BT44 micellar formulation will allow parenteral administration and help to alleviate the use of toxic excipients not only at its preclinical development phase but also potentially for future clinical use.

## Supporting Information Available

Synthesis of 2-*n*-pentyl-2-oxazoline monomer and triblock copolymers, DSC thermogram of three POx-based triblock copolymers, groups contributing toward HSPs determination, quantification of BT44 by HPLC, visual appearance of BT44 formulation, ^1^H NMR spectra of polymers and formulation, and DSC thermogram of the lyophilized formulation (PDF)
