# Supplementary material for: Biological Activity In Vitro, Absorption, BBB Penetration, and Tolerability of Nanoformulation of BT44:RET Agonist with Disease-Modifying Potential for the Treatment of Neurodegeneration
Source: Biomacromolecules. 2022 Oct 11;24(10):4348–65. doi: 10.1021/acs.biomac.2c00761 (PMC10565809; doi:10.1021/acs.biomac.2c00761)
Supplement: Supplementary file 1 — bm2c00761_si_001.pdf [file bm2c00761_si_001.pdf]

Supporting Information

Biological activity *In vitro*, absorption, BBB penetration and tolerability of nanoformulation of BT44: RET agonist with disease-modifying potential for the treatment of neurodegeneration

Malik Salman Haider[1, 6, ‡,*], Arun Kumar Mahato[2, ‡], Anastasiia Kotliarova[2], Stefan Forster[1], Bettina Böttcher[3], Philipp Stahlhut[4], Yulia Sidorova[2, *‡], Robert Luxenhofer[1,5‡]

[1]*Functional Polymer Materials, Chair for Advanced Materials Synthesis, Institute for Functional Materials and Biofabrication, Department of Chemistry and Pharmacy, Julius-Maximilians-University Würzburg, Röntgenring 11, 97070 Würzburg, Germany*

[2]*Laboratory of Molecular Neuroscience, Institute of Biotechnology, HiLIFE, University of Helsinki, 00014, Helsinki, Finland*

[3]*Biocenter and Rudolf Virchow Centre, Julius-Maximilians-University Würzburg, Haus D15, Josef-Schneider-Str. 2, 97080 Würzburg, Germany*

[4]*Department of Functional Materials in Medicine and Dentistry, Institute of Functional Materials and Biofabrication and Bavarian Polymer Institute, Julius-Maximilians-University Würzburg, Pleicherwall 2, 97070 Würzburg, Germany*

[5]*Soft Matter Chemistry, Department of Chemistry, and Helsinki Institute of Sustainability Science, Faculty of Science, University of Helsinki, PB 55, 00014 Helsinki, Finland*

[6]*University Hospital of Würzburg, Department of Ophthalmology, Josef-Schneider-Street 11, D-97080 Würzburg, Germany.*

Synthesis 1: 2-n-pentyl-2-oxazoline Monomer

Hexanenitrile	17.55 g	(180.58 mmol; 1.0 eq)
Ethanolamine	13.24 g	(216.70 mmol; 1.20 eq)
$ZnAc_2.(H_2O)_2$	0.99 g	(4.51 mmol; 0.025 eq)
Boiling point	78°C (17 mbar)	
Yield	13.6 g (53 % of colourless liquid)	

Synthesis 2: A-pPentOx-A triblock copolymer

Inititation	MeOTf	0.539 g	(3.29 mmol; 1 eq)
1st block	MeOx	9.80 g	(115.2 mmol; 35 eq)
2nd block	PentOx	9.29 g	(65.81 mmol; 20 eq)
3rd block	MeOx	9.80 g	(115.2 mmol; 35 eq)
Termination	1 molar aqueous NaOH solution was added		
Solvent	Benzonitrile	65 ml	
Yield	26 g of white powder 92 %		
GPC (HFIP)	M_n = 3.4 kg/mol; Đ = 1.19		
^{1}H-NMR	M_n = 8.1 kg/mol		

DSC of three ABA triblock copolymers

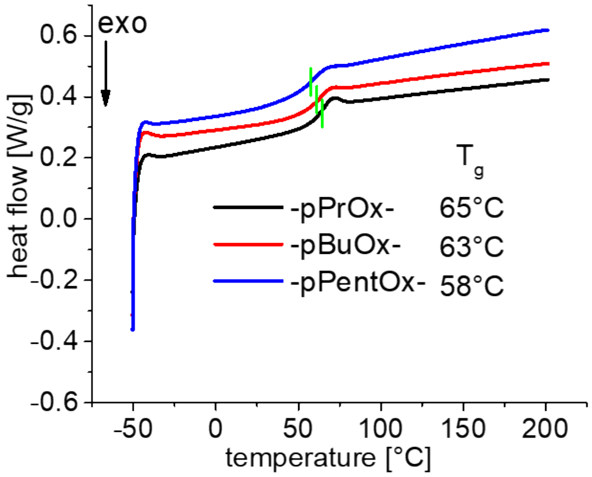

Figure S1: DSC thermogram of the three triblock copolymer used in this study. Heat flow occurring during the second heating (10 K/min) cycle, green vertical line indicating the glass transition points.

Groups contributing towards the HSPs determination

Table S1: Groups contributing towards the calculation of Hansen solubility parameters by Hoftyzer and Van Krevelen's method according to the equation no. 4, 5 & 6 as given in the main text. The sulfone group was split into its components due to unavailability of data in the reference table [1].

Groups	pPrOx	pBuOx	pPentOx	pMeOx	BT-44
CH_3-	1	1	1	1	1
-CH_2-	4	5	6	2	7
Phenyl	-	-	-	-	3
F	-	-	-	-	4
-O-	-	-	-	-	1
-CO-	1	1	1	1	1
>N-	1	1	1	1	3
SO_2 (-S/O-)	-	-	-	-	1/2
Ring	-	-	-	-	2

Quantification of BT44 by HPLC

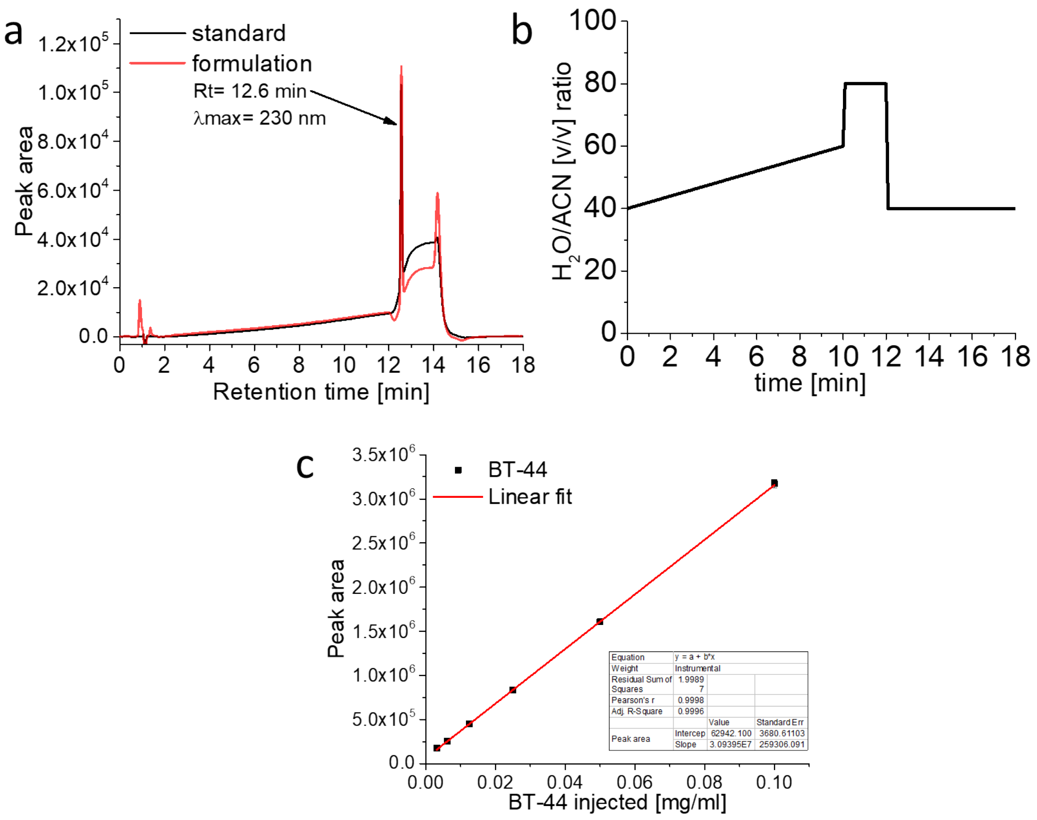

Figure S2: a) Normalized HPLC elugrams of BT44, standard dilution (black) and formulation (red) at λ_{max} of 230 nm. Rt = 12.6 min. b) The HPLC method with the water/acetonitrile gradient (%) with the flow rate of 1ml/min. c) HPLC calibration curve of known amounts of BT44 injected and corresponding linear fit (red curve).

Visual appearance of the BT44 formulations

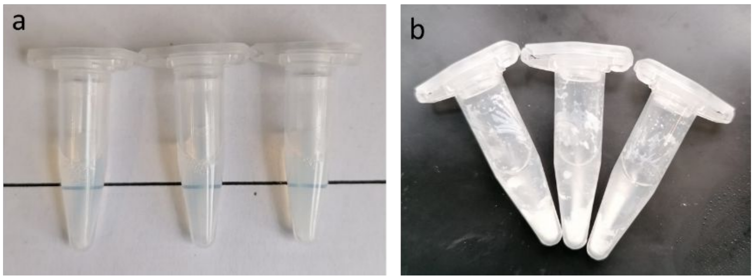

Figure S3: Visual appearance of the a) A-pPentOx-A/BT44 and b) A-pPrOx-A/BT44 formulation at 10/10 g/L polymer/BT44 feed (n=3).

^{1}H-NMR studies of the polymer and formulations

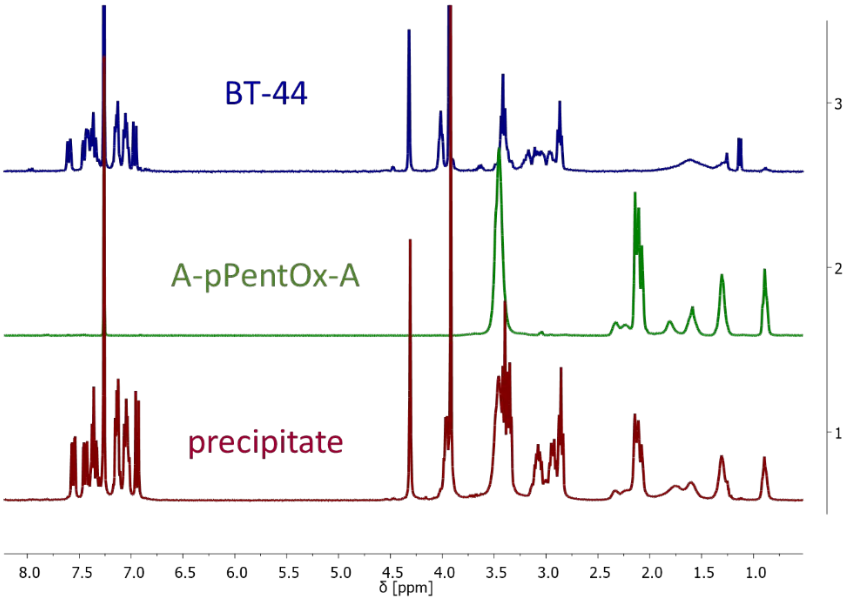

Figure S4: ^{1}H-NMR spectra (300 MHz, 298K) of the pure BT44 (blue), plain A-pPentOx-A polymer (green) and precipitate (red) formed during formulation development of A-pPentOx-A/BT44 (100/20 g/L). All the NMR spectra were obtained in $CDCl_3$.

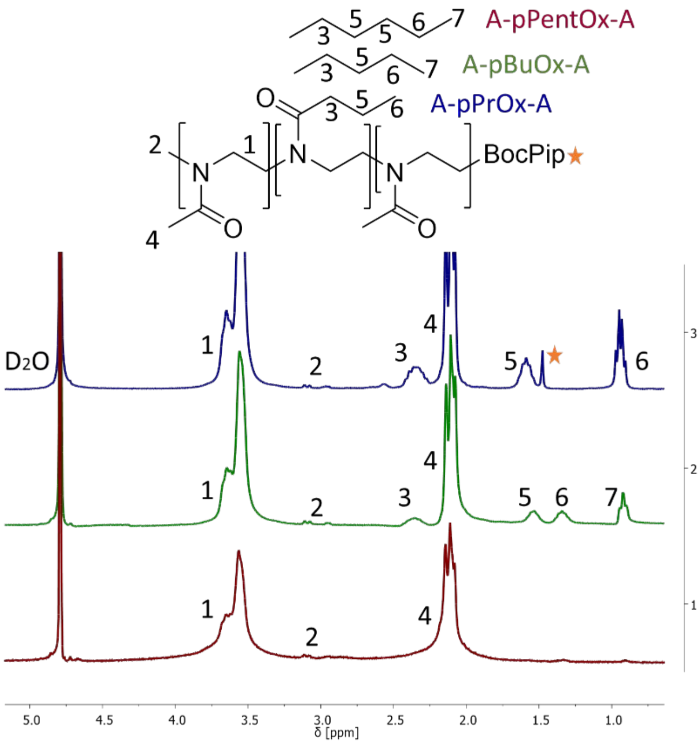

Figure S5: ^{1}H-NMR spectra (300 MHz, 298K) and chemical structures of the plain triblock copolymers i.e. A-pPrOx-A (blue), A-pBuOx-A (green) and A-pPentOx-A (red) in selective solvent D_2O at 10 g/L concentration with signal assignment of all major signals.

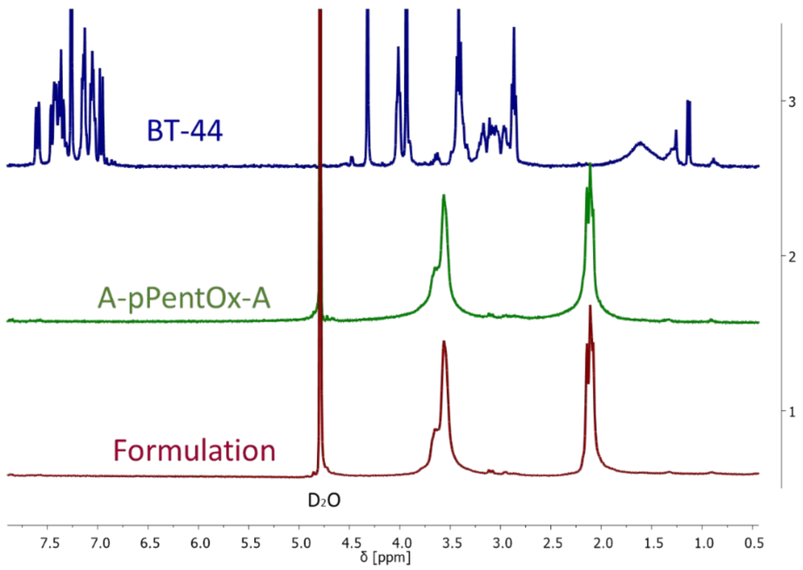

Figure S6: ^{1}H-NMR spectra (300 MHz, 298K) of the pure BT44 (blue) in $CDCl_3$, plain A-pPentOx-A polymer (green) and lyophilized formulation (A-pPentOx-A/BT44 100/20 g/L) collected in D_2O.

DSC studies of the formulations

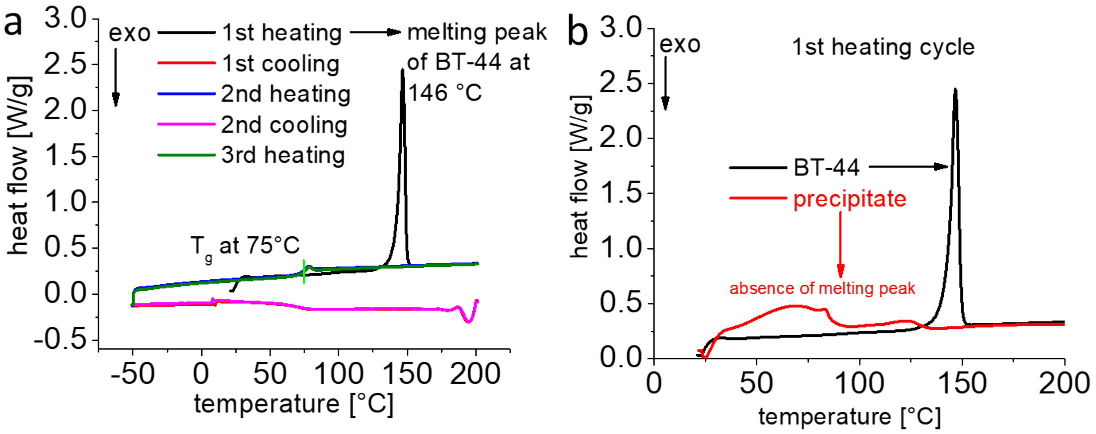

Figure S7: DSC thermogram of a) pristine BT44 with all the heating and cooling cycles (10 k/min). The sharp peak in first heating cycle at 146°C is the melting peak while the second and third heating cycles only exhibited the T_g (green vertical line at 75°C) and b) first heating cycle of pristine BT44 (black) and precipitated A-pPentOx-A/BT44 10/4 g/L formulation (red) at day 5 with sharp melting peak and absence of melting peak, respectively.

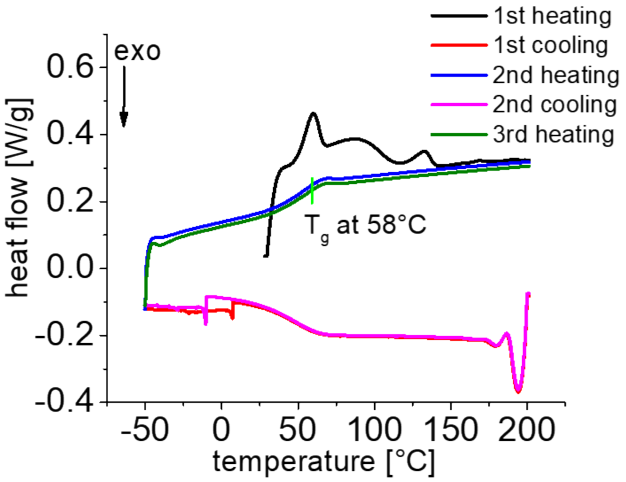

Figure S8: DSC thermogram with all the heating and cooling cycles (10 k/min) of A-pPentOx-A/BT44 100/20 g/L lyophilized formulation after 15 days of storage at room temperature. No melting peak of BT44 was observed in first heating cycle (black curve) and the second and third heating cycles only exhibited the T_g (green vertical line at 58°C) which corresponds to the T_g of A-pPentOx-A.

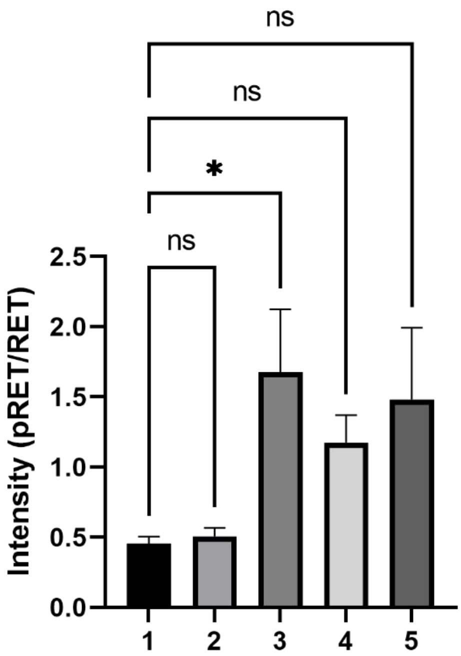

Figure S9: BT44 nanoformulation induces RET phosphorylation. The graph represents the quantification of RET phosphorylation level. The bars 1, 2, 3, 4, and 5 are VEH, Polymer, Formulation, BT44 and GDNF, respectively. *$p < 0.05$ RM ANOVA with Dunnett's post hoc test. Data are presented as means ± SEM, n = 2.

References:

1. Fedors, R.F., *A method for estimating both the solubility parameters and molar volumes of liquids.* Polymer Engineering & Science, 1974. **14**(2): p. 147-154.
